# Supplementary material for: Hospital at Home care for older patients with cognitive impairment: a protocol for a randomised controlled feasibility trial
Source: BMJ Open. 2018 Mar 27;8(3):e020332. doi: 10.1136/bmjopen-2017-020332 (PMC5875621; doi:10.1136/bmjopen-2017-020332)
Supplement: Author's manuscript [file bmjopen-2017-020332.draft_revisions.pdf]

BMJ Open

BMJ Open is committed to open peer review. As part of this commitment we make the peer review history of every article we publish publicly available.

When an article is published we post the peer reviewers' comments and the authors' responses online. We also post the versions of the paper that were used during peer review. These are the versions that the peer review comments apply to.

The versions of the paper that follow are the versions that were submitted during the peer review process. They are not the versions of record or the final published versions. They should not be cited or distributed as the published version of this manuscript.

BMJ Open is an open access journal and the full, final, typeset and author-corrected version of record of the manuscript is available on our site with no access controls, subscription charges or pay-per-view fees (http://bmjopen.bmj.com).

If you have any questions on BMJ Open's open peer review process please email editorial.bmjopen@bmj.com

Hospital at Home care for older patients with cognitive impairment: A protocol for a randomized controlled feasibility trial

Journal:	*BMJ Open*
Manuscript ID	bmjopen-2017-020332
Article Type:	Protocol
Date Submitted by the Author:	30-Oct-2017

Complete List of Authors:	Pouw, Maaike; Universitair Medisch Centrum Groningen, Geriatrics; Martini Ziekenhuis, Internal Medicine<br>Calf, Agneta; Universitair Medisch Centrum Groningen, Geriatrics<br>van Munster, Barbara; Universitair Medisch Centrum Groningen, Geriatrics; Gelre Ziekenhuizen, Geriatrics<br>ter Maaten, Jan; Universitair Medisch Centrum Groningen, Internal Medicine, Emergency Medicine<br>Smidt, Nynke; University of Groningen, Epidemiology<br>De Roolj, Sophia; University Medical Center Groningen, Geriatrics

Keywords:	Dementia < NEUROLOGY, Organisation of health services < HEALTH SERVICES ADMINISTRATION & MANAGEMENT, GERIATRIC MEDICINE, Delirium & cognitive disorders < PSYCHIATRY, INTERNAL MEDICINE

SCHOLARONE™ Manuscripts

Public title

Hospital at Home care for older patients with cognitive impairment and an acute medical illness

Scientific title

Hospital at Home care for older patients with cognitive impairment: A protocol for a randomized controlled feasibility trial

Maaike A Pouw,[1,2] Agneta H Calf,[1] Barbara C van Munster,[1,3] Jan C ter Maaten,[4] Nynke Smidt,[1,5] & Sophia E de Rooij [1]

Acronym

H@H

Abstract

INTRODUCTION: An acute hospital admission is a stressful life event for older people, particularly for those with cognitive impairment. The hospitalisation is often complicated by hospital-associated geriatric syndromes, including delirium and functional loss, leading to functional decline and nursing home admission. Hospital at Home care aims to avoid hospitalisation-associated adverse outcomes in older patients with cognitive impairment by providing hospital care in the patient's own environment.

METHODS AND ANALYSIS: This randomized, non-blinded feasibility trial aims to assess the feasibility of conducting a randomized controlled trial in terms of the recruitment, use and acceptability of Hospital at Home care for older patients with cognitive impairment. The quality of care will be evaluated and the advantages and disadvantages of the Hospital at Home care program compared to usual hospital care. Eligible patients will be randomized either to Hospital at Home care in their own environment or usual hospital care. The intervention consists of hospital level care provided at patients' homes, including visits from health care professionals, diagnostics (laboratory tests, blood cultures) and treatment. The control group will receive usual hospital care. Measurements will be conducted at baseline, during admission, at discharge and at three and six months after the baseline assessment.

ETHICS AND DISSEMINATION: Institutional ethics approval has been granted. The findings will be disseminated through public lectures, professional and scientific conferences, as well as peer-reviewed journal articles. The study findings will contribute to knowledge on the implementation of Hospital at Home care for older patients with cognitive disorders. The results will be used to inform and support strategies to deliver eligible care to older patients with cognitive impairment.

TRIAL REGISTRATION: This study was registered with the NTR registry (NTR6581) on 26 July 2017.

Keywords: Dementia, Cognition disorders, Central Nervous System Diseases, Aged, Home Care Services; Hospital-Based, Hospitalization, Randomized Controlled Trials, Hospital at Home

For peer review only - http://bmjopen.bmj.com/site/about/guidelines.xhtml

Strengths and limitations of this study

- This study addresses patients with cognitive impairment, a population who might benefit most of the Hospital at Home care concept, but is often excluded from participation in scientific research.
- As a feasibility trial does not show effectiveness, it will support the implementation of Hospital at Home care and a future trial
- This study is clinically relevant as it investigates patient centred care instead of hospital centred care

Introduction

Background

An acute hospital admission is a stressful life event, particularly for older people. In addition to the stress of an acute illness, the hospital admission itself contributes to this stress.[1] Older hospitalized patients are often deprived of sleep, and they spend an average of 20 of every 24 hours in bed, they become poorly nourished, and experience sensory deprivation or overstimulation, resulting in confusion.[2-5] These adverse effects of hospitalization contribute to the occurrence of geriatric conditions, such as delirium, functional decline, falls, incontinence, hospital acquired infections and pressure injuries.[6-9] Adverse effects of hospitalization occur more easily in older people, particularly in those who are already frail, a growing portion of the worldwide ageing population.[10,11] Frailty is a state of increased vulnerability to external stressors resulting from aging-associated declines in reserve and function across multiple physiologic systems.[10] Cognitive impairment (i.e., dementia) is an important contributor to frailty in older people.[12] Cognitively impaired older people are more likely to become hospitalised and once admitted, they experience longer stays than their peers without cognitive impairment.[13-15] The combination of hospitalization and cognitive impairment in older people is associated with further functional and cognitive declines and higher mortality rates, and it leads to more discharges to long-term care facilities.[16,17] The prevalence and worldwide burden of cognitive impairment will continue to increase as the average life expectancy increases.[18] The total number of people with cognitive impairment is estimated to be 75.6 million in 2030 and will nearly triple in 2050 to 135.5 million.[19] An increase in the number of hospital admissions of older people with cognitive impairment and an increase in number of hospitalisation-associated adverse outcomes are therefore to be expected.

Besides adverse outcomes of hospitalization, many older people and their caregivers do not necessarily desire a hospital admission in case of an acute illness or exacerbation of a chronic illness. Fried et al. (2000) have studied the preferences of community-dwelling persons 65 years of age and

For peer review only - http://bmjopen.bmj.com/site/about/guidelines.xhtml

older who were hospitalized with a primary diagnosis of congestive heart failure, chronic obstructive pulmonary disease or pneumonia. The authors reported that over 50% of older patients preferred to receive hospital treatment at home, because they felt that their homes were more comfortable.[20] In the treatment preferences of seriously ill patients 60 years of age and older, the likelihood of cognitive and functional impairment as an adverse outcome of the treatment was weighed in the decision-making process. There was a substantial decrease in the number of participants who opted for treatment if the likelihood of impairment after treatment was 50% or higher.[21]

Hospital at Home care could provide an effective alternative to inpatient care for a select group of elderly patients now requiring hospitalisation. Hospital at Home care is coordinated, multidisciplinary care in the homes of people who would otherwise be admitted to the hospital. Hospital at home care is an accepted alternative to inpatient hospital level care in several countries (e.g., the United States, Australia, Italy and the United Kingdom) but not yet in the Netherlands.[22] Since the 1990s, Hospital at Home has been evaluated in (older) persons with various acute medical conditions, such as heart failure, exacerbations in chronic obstructive pulmonary disease (COPD) and infections (e.g., cellulitis, pneumonia).[22] In a systematic review comparing alternative strategies to inpatient hospitalization, lower or equal mortality rates and return hospitalization rates (i.e., subsequent admissions after discharge) were found for Hospital at Home care, and there was a positive effect on patient and caregiver satisfaction.[22,23] Only one trial conducted in Italy included 109 patients with cognitive impairment (i.e. dementia), Tibaldi et al. reported a positive effect of a Hospital at Home intervention on behavioural disturbances and caregiver stress in patients with dementia.[24] Whether Hospital at Home care provides a suitable alternative with regard to other outcomes as patient satisfaction, quality of care, hospitalisation-associated adverse events and costs in older people with cognitive impairment remains unclear and further research is needed. Therefore, our primary aim is to investigate the feasibility of a Hospital at Home care program for older patients with cognitive impairment in terms of the patient recruitment, use and acceptability, and secondly to investigate the advantages and disadvantages of Hospital at Home care compared to usual hospital care from different perspectives.

Objectives of this study are:

1) To assess the participation rate of the Hospital at Home trial among patients 65 years and older with cognitive impairment, acute illness, and emergency hospital admission. What are the reasons for non-participation?

2) To assess the differences in the quality of care provided in the Hospital at Home care group and the usual hospital care group with regard to geriatric syndromes, institutionalization, mortality, total

For peer review only - http://bmjopen.bmj.com/site/about/guidelines.xhtml

days with urinary catheter, length of stay (in the hospital or in Hospital at Home care) and timing/intensity of the contact with health care professionals.

3) To assess the potential advantages and disadvantages of Hospital at Home care and usual hospital care for the patients, caregivers and Dutch medical health system.

Methods and analysis

Trial design

The design is a randomized controlled feasibility trial and will utilize a process evaluation. This study will be conducted at the medical emergency department (ED) of the academic hospital of the University Medical Center of Groningen in the Netherlands and will evaluate cognitively impaired older patients who are in need of acute hospital care. Figure 1 shows the trial design summary. Participants will be randomized to either Hospital at Home care or usual hospital care in a 4:1 ratio, respectively. Patients will be randomized using a computerized random number generator (http://www.randomization.com), including block randomization.

An independent research nurse who is not involved in the patient care will complete the baseline assessment and allocate the participants (using sealed sequenced envelopes) into the Hospital at Home care (intervention) or usual hospital care group (control). The research nurse will not be aware of the randomization method. The participants, health care professionals and research staff will not be blinded to the intervention. The reporting of the design of this trial protocol is in accordance with the SPIRIT 2013 statement for clinical trial protocols.[25]

only

For peer review only - http://bmjopen.bmj.com/site/about/guidelines.xhtml

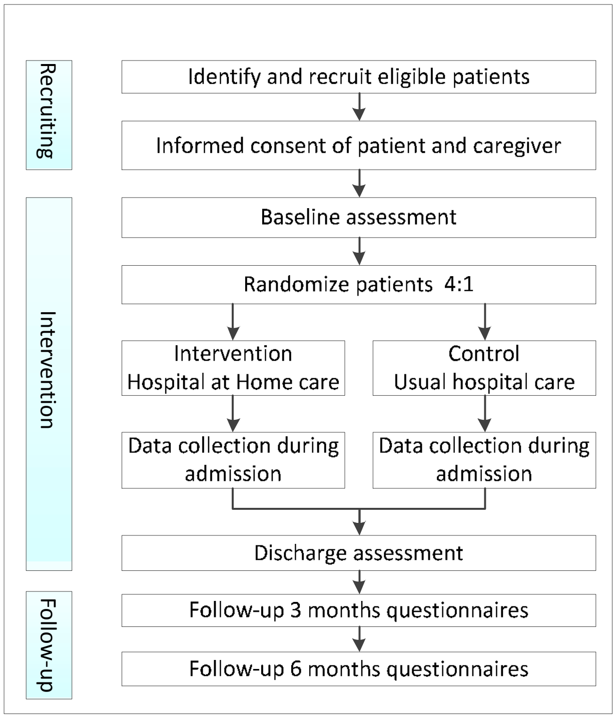

Figure 1: Flowchart of trial design summary

Study population

Patients 65 years of age and older who are admitted to the medical emergency department (ED) will be identified by the ED staff as potential eligible patients. Subsequently, the ED staff will inform the research nurse. The research nurse will complete the eligibility criteria checklist (table 1). The research nurse will ask the patient and their informal caregiver (i.e., partner, child, relative, friend) for their willingness to participate in the study and to provide written informed consent. The patient and informal caregiver will need to both sign the informed consent form before the patient can participate in the H@H trial.

If the participant lacks the capacity to consent (mentally incapacitated), and an informal caregiver (i.e., partner, child, friend) is present, this informal caregiver will be asked to act as a personal consultee. The personal consultee will determine whether he or she believes that participation in the study would be in accordance with the values and interests of the individual and will subsequently sign the patient's informed consent form.

For peer review only - http://bmjopen.bmj.com/site/about/guidelines.xhtml

Table 1 Patient eligibility criteria for participation in Hospital at Home trial

Inclusion criteria	Exclusion criteria
▪ Age 65 years of age and older<br>▪ Cognitive impairment, i.e. dementia, delirium or other cause of cognitive impairment, and either:<br>○ previously diagnosed or documented in the medical records or<br>○ identified by the ED-clinician (e.g., with the 4AT-test and/or Six item cognitive impairment test)[26-28]<br>▪ Presented at the ED with a defined acute illness<br>▪ Required hospital admission, according to the attending ED physician but not expected to require emergency interventions<br>○ Modified Early Warning Score (MEWS) ≤ 2 points[29-31]<br>▪ Living in hospital's catchment area (< 25 km)<br>▪ Informal caregiver is present and able to understand and perform instructions and consented to participate in the trial<br>▪ Home suitable for Hospital at Home care (available informal caregiver, running water, adequate heating, safety) [28]	▪ Previously enrolled<br>▪ Hospitalized within the seven days preceding ED presentation<br>▪ Nursing home residents or awaiting a nursing home place on an active waiting list (excluding so-called sleeping waiting list candidates)[27]<br>▪ Additional care needed<br>○ Required surgical assessment<br>○ Suspected acute coronary syndrome or cardiac arrythmia[31]<br>○ Dialysis dependent patients[31]<br>○ Expected terminal events[31] or in need of diagnostic or palliative care due to oncological or haematological illness

ED= Emergency department

Sample size of study population

Based on the numbers available from the emergency department (ED) of the University Medical Center Groningen, the Netherlands, we calculated that an average of 3990 older patients 65 years and over is admitted to our medical ED each year. The Hospital at Home trial will be introduced during working hours, which provides an estimated 1900 patients per year. Not all 1900 patients will be eligible for study inclusion. Based on screening of ED medical records, approximately 15% of the patients meet the eligibility criteria for Hospital at Home care, resulting in 285 eligible persons per year. In recent randomized controlled trials (RCTs) of Hospital at Home care in Italy, 54% and 57% of the eligible patients was willing to participate and gave informed consent.[32,33] We presume a similar consent rate of 50%, as described in these previous clinical trials, and expect for 143 patients to be included.

Study procedures

After (written) informed consent is obtained, the participants will complete two brief cognitive tests, and the participant and caregiver will complete the baseline assessment. Subsequently, randomization takes place to either (a) the Hospital at Home care-intervention, translocation of care from the hospital to a participants' home or (b) the control group, usual hospital care. All care will be delivered according to hospital protocols, current regulations and guidelines and, if needed,

For peer review only - http://bmjopen.bmj.com/site/about/guidelines.xhtml

described in the standard operating procedures (SOPs). If no informed consent is given by either the patient or the caregiver, the reasons for non-participation, date of birth, sex of the patient and the relationship between patient and caregiver will be reported.

Intervention

Hospital at Home care

Hospital at Home care will be delivered by a multidisciplinary team consisting of a physician, nurse and physiotherapist. Depending on the participants' needs, other disciplines (e.g., a dietician, occupational therapist or social services) can be involved in the Hospital at Home care. The day-to-day care will be provided by the nurse and physician visiting the participant. The Hospital at Home care team works under the responsibility of the medical specialist in the hospital, and 24/7 consultation of the expertise and services of the hospital is part of the protocol.

The participants allocated to Hospital at Home care will receive hospital level care in their own homes. After a stay of one night in the hospital, while the Hospital at Home care arrangements are being made, the participant will be transferred home and receive Hospital at Home care. The Hospital at Home nurse is responsible for the day-to-day care and will be present upon arrival of the participant at his/her residence. Hospital at Home care is described in the care protocols including SOPs and could include intravenous therapy (e.g., antibiotics, fluid, and/or diuretics), oxygen therapy, and/or nebulizer, indwelling urine catheter or a nasopharyngeal food tube. After the care intake and a period of direct nursing supervision, the participant will receive intermittent nursing visits daily (starting with 3x/day). The Hospital at Home physician will make a home visit every day (excl. weekends). The Hospital at Home physician and nurse will be available for emergency visits. The participant will receive a medical alert device in the house, with a 24/7 connection to an on-call service. Alert instructions will be explained to the participant and caregiver. A physiotherapist will visit the participant at home to evaluate any problems with balance and/or walking and immobility. The Hospital at Home team works under the supervision of the hospital medical specialist. Daily screenings and measurements will be recorded in a Hospital at Home record, which stays with the participant. Diagnostic procedures and therapeutics that cannot wait and are not available at home, such as endoscopy or CT-scan, will be arranged through brief visits to the hospital. The participant will be 'admitted' to the Hospital at Home care for as long as indicated.

Discharge from Hospital at Home

If the participant recovers to such an extent that hospital level care is no longer needed, the participant will be discharged from the Hospital at Home care program, similar to the discharge procedure when the participant would receive usual hospital care. Hospital at Home care will end

For peer review only - http://bmjopen.bmj.com/site/about/guidelines.xhtml

with discharge planning with the participant, family, Hospital at Home physician and nurse. The discharge plan includes follow-up appointments (e.g., at the hospital or general practitioner), information on medication, warning signs and symptoms and an on-going management plan. All hospital-related care equipment will be removed from the participants' house, and arrangements with homecare agencies and/or paramedical staff will be reviewed and adjusted to the current situation.

Hospital care as usual

Participants allocated to the control arm will be admitted to a hospital ward and receive usual hospital care. After admission and intake on the ward, the participant will receive intermittent visits from the ward nurse multiple times a day. The ward physician will visit once every day (excl. weekends), with extra visits provided if needed. An emergency alert device, through which nurses and physician can be contacted, will be placed next to the bed. A physiotherapist will visit the participant at the ward to address problems with balance and/or walking and immobility. Depending on the participants' needs, other disciplines (e.g., a dietician, occupational therapist or social services) can be involved in the hospital care. The medical record is the hospital record and additional H@H research forms will be added to this record for research purposes. The participant will be admitted to the hospital for as long as indicated.

Hospital discharge

If the participant recovers to such an extent that hospital level care is no longer needed, the participant will be discharged from the hospital after discharge planning with the participant, family, physician and nurse. The discharge plan includes follow-up appointments (e.g., at the hospital or general practitioner), information on medication, warning signs and symptoms and an on-going management plan. Arrangements with homecare agencies and/or paramedical staff will be reviewed and adjusted to the current situation.

Follow-up

At three and six months following randomization, all participants will be contacted for an interview by telephone[34] or a face-to-face interview, if needed. The participants will be allowed to receive support with these questions from their relatives or informal caregivers. An interview will require a maximum of 30 minutes. In case of institutionalization or mortality, this event will be recorded. Additionally, information on hospital readmission and length of stay will be collected from the hospital administration system and health insurers. Mortality and nursing home placements will be collected from registries from the general practitioner and municipalities.

For peer review only - http://bmjopen.bmj.com/site/about/guidelines.xhtml

Timing of measurements and outcome measures

Data will be collected at baseline at the emergency department, during admission (in Hospital at Home or hospital), at discharge and at three and six months following randomization, plus or minus two weeks. An overview of the timing of measurements and outcome measures are shown in Table 2.

Feasibility

For the participation rates, the proportion of participants per step will be calculated. The reasons for non-participation and data concerning the characteristics of non-participants will be collected. We consider the participation rate feasible when it is similar to the participation rate as is described in previous RCTs and around 50% of the eligible patients will consent to participate.[32,33] Quality of care will be measured by collecting data on patient, institutionalization (e.g., to the hospital or nursing home), mortality, ADL-functioning, prevalence of hospitalisation-associated geriatric syndromes, the length of stay in the hospital or Hospital at Home care program and contact with health care professionals. The study is considered feasible if the quality of care of Hospital at Home care on these measurements is non- inferior to usual hospital care.

For peer review only - http://bmjopen.bmj.com/site/about/guidelines.xhtml

Table 2 Overview of the content and description of outcome measures and timing of measurements

	Description and instrument	Timing of measurements: screening	baseline	daily	discharge	3 months	6 months
Degree of illness based on physiological parameters	Vital signs alarm score; Modified Early Warning Score, [35]	R					
Cognitive impairment	4AT-test for delirium* Six item cognitive impairment test for cognitive impairment*,[26,36]	R					
Socio-demographics	Date of birth, nationality, household composition, marital status, highest level of education		R				
Health status	Charlson co-morbidity index*,[37]		R				
Identifying at-risk patients	Safety management system patient screening (VMS),[38]		R				
Functional status	Activities of daily living, modified Katz-ADL index score,[39]		R		R	R	R
Health status	EQ-5d-5l*,[40]		R		R	R	R
(Health-related) quality of life, well-being	Icepop capability measure for Older people (ICECAP-O)*[41]		R		R	R	R
Caregiver burden	Self-rated burden scale*, caregiver strain index*,[42,43]		C		C	C	C
Medical consumption	Imta Medical Consumption Questionnaire (imcq)*,[44]		P,C			P,C	P,C
Hospitalisation-associated geriatric syndromes	Infections, falls, pressure injuries, in case of delirium; delirium observation scale score (DOSS)[45] and use of physical or chemical restraints, total days with a urinary catheter			N			
Nutrition	Malnutrition Universal Screening Tool (MUST),[46] food intake, fluid intake			N			
Pain	Numeric Rating Scale-score (NRS) for pain [47]			N			
Health perception	(Rotterdam) symptom checklist*[48]			N			
Immobility	Hierarchical assessment of balance and mobility (HABAM)[49]			Ph			
Satisfaction with care	Client Satisfaction Questionnaire 8 (CSQ-8)*, care evaluation question*,[50,51]				P<br>P,C,<br>N,D		
Mortality	Mortality at 30 days, 3 months and 6 months after baseline*				N	R	R
(Re)admission hospital	Length of stay, readmission rate at 30 days, 3 months and 6 months after baseline*				N	R	R

Assessed by: P= Participant, C=Caregiver, N= Nurse, D= Doctor, R= Research nurse, Ph=Physiotherapist.
(*)= All assessments marked by an asterisk are extra for trial purposes and are not part of the medical treatment

Other outcomes measures

Advantages and disadvantages of the Hospital at Home care program will be assessed through multiple instruments and questionnaires. The instruments are validated and used in community-dwelling older patients with cognitive disorders. Additional data will be collected on the time spent at home (home-time); total number of days alive and out of the hospital or a skilled nursing facility in the 6 months following the randomization at the emergency department,[52] the number of transfers (home ↔ hospital) and the number of health care professionals involved.

For peer review only - http://bmjopen.bmj.com/site/about/guidelines.xhtml

Cost data will be collected, as described by Drummond et al., including the costs to the health care system, patients and families, and other sectors.[53] The volume of care use will be extracted from hospital files and combined with the reference cost values, as provided by the cost guidance module of the Dutch National Health Care Institute.[54]

Process evaluation

A process evaluation will be conducted as part of the feasibility study to understand the barriers and facilitators to participate and to gain an understanding of the experiences and perceptions of Hospital at Home care of participants and health care professionals. From all eligible patients who declined to participate, data concerning the patient characteristics and reasons for non-participation will be collected. At the end of the trial, a representative sample of participants and/or their informal caregivers will be invited for an interview to evaluate their experiences receiving Hospital at Home care. The interviewer will not be a an active member of the research team or involved in day-to-day care and will explore independently how the participants perceived Hospital at Home care, including the contact with the health professionals and the impact of Hospital at Home care on their lives and their caregivers' personal lives. In case of participant dropout, efforts will be made to obtain an understanding of why the participants did not complete the trial.

In addition, a representative sample of health care professionals, consisting of physicians and nurses working in the Emergency Department, physicians and nurses providing the Hospital at Home care, and general practitioners, will be asked to participate in a face-to-face interview. The health care professionals will be asked about their experiences and opinions about the H@H-trial and Hospital at Home care. All interviews will be transcribed verbatim, and a framework analysis will be used as the method of qualitative data analysis.[55]

Data management

All data will be entered in an electronic trial-specific database, with the participants identified by a unique trial number. Confidentiality of participant information will be maintained throughout the trial. Information can only be traced to the participants by designated researchers. The database will be stored and maintained by Castor Electronic Data Capture, compliant with GCP guidelines and the European Data Protection Directive (Castor Electronic Data Capture, Ciwit BV, Amsterdam, the Netherlands, 2017). Data will be stored for a maximum period of 15 years after the study has ended, according to Dutch law.[56]

For peer review only - http://bmjopen.bmj.com/site/about/guidelines.xhtml

Statistical analysis

The participant flow diagram, according to CONSORT guidelines,[57] will provide a summary of the recruitment and declination rates in percentage (%) at baseline, discharge and three- and six-month follow-ups. Distributions of the data at baseline, discharge and three and six months after randomization will be explored, with unusual values noted and explained. Variables will be summarized as the n (%), mean (standard deviation) or median (interquartile range) for each group, to characterize the sample and search for any imbalances. The percentages, means and standard deviation, and medians and interquartile ranges will be calculated to describe the quality of care and the advantages and disadvantages of Hospital at Home care at baseline, discharge and three- and six-month follow-ups.

Monitoring and participant safety

Although, the H@H trial is considered to be a low risk trial, the participant safety will be monitored by an independent Data Monitoring Committee (DMC). The DMC will consist of two members: an experienced clinician and an epidemiologist. Members of the DMC are independent of the trial and will discuss each individual participant with serious adverse events. The DMC will receive and review the serious adverse events and evaluate the risk involved with negative outcomes. The DMC is authorized to make recommendations to temporary put on hold or ending the study prematurely when participant safety is an issue, based on their findings. All serious adverse events will be reported to the principal investigator within 24 hours of knowledge of the event and then subsequently reported to the Dutch portal for medical research involving human subjects.

Discussion

Reducing unwanted hospital admissions in older patients with cognitive impairments and facilitating patient-centred care in a patient's preferred location is a goal worthy of pursuing. This goal aligns with the tenet of the current Dutch government and the advice provided by the Dutch Council for the Environment and Infrastructure: to actively promote and enable people to live independently in their own homes for as long as they desire.[58] Previous trials and a recent review have confirmed that alternative management strategies for low-risk patients with acute medical conditions conventionally treated through hospitalization exist with positive impact on patient satisfaction, are effective and can be safely achieved in lower cost settings.[22,23,59]

For peer review only - http://bmjopen.bmj.com/site/about/guidelines.xhtml

Introducing a Hospital at Home care trajectory in the Netherlands is incited by the principles of value-based health care: improving the patients experience of care and as a result of this process reducing the costs.[60] All countries with an ageing population experience pressure, in terms of shortage of (emergency) hospital beds and rising healthcare costs. Hospital at Home care could be shown beneficial in facilitating higher valued care for patients and their caregivers without additional costs. Benefit should be measured in other outcomes than clinical indicators such as mortality. To illustrate, one of the outcomes of a future RCT could be the time spent at home. Time spent at home has been defined as the total number of days alive and out of the hospital or a skilled nursing facility in the 6 months after hospital admission.[52] It has been used as a primary outcome in a follow-up study of older patients with acute hospital admissions, and has been demonstrated to be of more importance in older patients.[61-63] Evaluation of time spent a home in this feasibility study could support estimating a sample size based on a patient-relevant outcome in a future RCT.

This study will be the first to investigate the feasibility of providing acute hospital care at home for older patients with cognitive impairment in the Netherlands. Studying Hospital at Home care and identifying the barriers and facilitators will support the implementation of Hospital at Home care and break new ground for a future RCT investigating the (cost-)effectiveness.

Ethics and Dissemination

This study was approved by the Medical Research Ethics Committee of the University Medical Center Groningen in April 2017, reference number: 2016.686 The trial will be conducted in accordance with the Declaration of Helsinki 1996, principles of good clinical practice and the University Medical Center of Groningen Research Code. Any protocol amendments will be submitted to the ethics committee. A register of the protocol amendments will be available in the study protocol.

The results of the trial will be reported according to the CONSORT guidelines and will contribute to knowledge of the implementation of Hospital at Home care and patient-centred acute care for older patients with cognitive impairment. The study will also contribute to the knowledge of the transmural cooperation and costs of providing care, in terms of the translocation of hospital care to home. Regularly updates will be published on the study website and in newsletters. Conferences and meetings will be held for all involved health care professionals. Participants who requested information on the study will be sent a lay summary. A publication policy will be agreed upon with co-applicants. The study findings will be published in relevant peer-reviewed journals.

For peer review only - http://bmjopen.bmj.com/site/about/guidelines.xhtml

Authors' affiliations
[1] Department of Geriatrics, University Medical Center Groningen, University of Groningen, Groningen, the Netherlands
[2] Department of Internal Medicine, Martini Hospital, Groningen, The Netherlands.
[3] Department of Geriatrics, Gelre Hospitals, Apeldoorn, The Netherlands.
[4] Department of Internal Medicine, Emergency Department, University Medical Center Groningen, University of Groningen, Groningen, The Netherlands
[5] Department of Epidemiology, University of Groningen, University Medical Center Groningen, Groningen, The Netherlands.

Acknowledgements The authors would like to thank all care professionals of Alzheimer Nederland, Espria, Icare, TSN, Buurtzorg, umcGroningen thuis, MCZ, Netwerk Dementie Groningen, Netwerk Dementie Drenthe, Gemeente Groningen, WIJ Groningen, Zorgbelang, Healthy Aging Network Northern Netherlands, Stichting Effectieve Ouderenzorg, ZonMW, Deltaplan Dementie, Nationaal Programma Ouderenzorg, Zorg innovatie Forum and the elderly of Denktank 60+ Noord for their time, critical appraisal and advise.

Contributors MP drafted the manuscript. All authors were involved in making substantial contributions to the conception and design of the protocol and the critical revision of the manuscript for publication. All authors approved the final version to be published.

Funding This study is independent research funded by ZonMw (project Memorabel, Deltaplan Dementie), project number #733050401 (contact: memorabel@zonmw.nl). Additional funding is provided by the Dutch Ministry of Health, Welfare and Sport (Ministerie van Volksgezondheid, Welzijn en Sport) and the University Medical Center Groningen.

Disclaimer The views and opinions expressed in this paper are those of the authors and not necessarily those of the UMCG, Memorabel, Deltaplan Dementie or ZonMW.

Competing interests All authors have declared that they have no competing interests

Ethics approval for this study has been obtained from the Medical Research Ethics Committee of the University Medical Center Groningen April 2017 (ref: 2016.686).

Recruitment status is pending; participants are not yet being recruited or enrolled. Anticipated date of enrolment of the first participant is December 2017.

Corresponding author: m.a.pouw@umcg.nl. University Center of Geriatric Medicine, University Medical Center Groningen. Hanzeplein 1, Postbus 30 001, 9700 RB Groningen

Scientific inquiries: prof Sophia E.de Rooij, s.e.j.a.de.rooij@umcg.nl, telephone number 050-3613921, Hanzeplein 1, Postbus 30 001, 9700 RB Groningen. University of Groningen, University Medical Center Groningen, University Center for Geriatric Medicine, Groningen, The Netherlands.
Generic email address hospitalathome@umcg.nl.

Abbreviations

ADL: Activities of Daily Living
CT: Computer Tomography
DMC: Data Monitoring Committee
ED: Emergency Department
H@H: Hospital at Home
RCT: Randomized Controlled Trial
UMCG: University Medical Center Groningen

References

(1) Krumholz HM. Post-Hospital Syndrome - A Condition of Generalized Risk. N Engl J Med 2013 01/10;368(2):100-102.

For peer review only - http://bmjopen.bmj.com/site/about/guidelines.xhtml

(2) Missildine K, Bergstrom N, Meininger J, Richards K, Foreman MD. Sleep in Hospitalized Elders: A Pilot Study. Geriatr Nurs 2010 0;31(4):263-271.

(3) Brown CJ, Redden DT, Flood KL, Allman RM. The underrecognized epidemic of low mobility during hospitalization of older adults. J Am Geriatr Soc 2009 Sep;57(9):1660-1665.

(4) Sullivan DH, Sun S, Walls RC. Protein-energy undernutrition among elderly hospitalized patients: a prospective study. JAMA 1999 Jun 2;281(21):2013-2019.

(5) Creditor MC. Hazards of hospitalization of the elderly. Ann Intern Med 1993;118(3):219-223.

(6) Inouye SK, Studenski S, Tinetti ME, Kuchel GA. Geriatric Syndromes: Clinical, Research, and Policy Implications of a Core Geriatric Concept. J Am Geriatr Soc 2007;55(5):780-791.

(7) Mudge AM, Banks MD, Barnett AG, Blackberry I, Graves N, Green T, et al. CHERISH (collaboration for hospitalised elders reducing the impact of stays in hospital): protocol for a multi-site improvement program to reduce geriatric syndromes in older inpatients. BMC Geriatrics 2017;17(1):11.

(8) Covinsky KE, Pierluissi E, Johnston CB. Hospitalization-associated disability: "She was probably able to ambulate, but I'm not sure". JAMA 2011 Oct 26;306(16):1782-1793.

(9) Zisberg A, Shadmi E, Gur-Yaish N, Tonkikh O, Sinoff G. Hospital-associated functional decline: the role of hospitalization processes beyond individual risk factors. J Am Geriatr Soc 2015 Jan;63(1):55-62.

(10) Fried LP, Tangen CM, Walston J, Newman AB, Hirsch C, Gottdiener J, et al. Frailty in older adults: evidence for a phenotype. J Gerontol A Biol Sci Med Sci 2001 Mar;56(3):M146-56.

(11) Clegg A, Young J, Iliffe S, Rikkert MO, Rockwood K. Frailty in elderly people. The Lancet 2013 3/2–8;381(9868):752-762.

(12) Ferrucci L, Guralnik JM, Studenski S, Fried LP, Cutler GB, Walston JD, et al. Designing Randomized, Controlled Trials Aimed at Preventing or Delaying Functional Decline and Disability in Frail, Older Persons: A Consensus Report. J Am Geriatr Soc 2004;52(4):625-634.

(13) Glover A, Bradshaw LE, Watson N, Laithwaite E, Goldberg SE, Whittamore KH, et al. Diagnoses, problems and healthcare interventions amongst older people with an unscheduled hospital admission who have concurrent mental health problems: a prevalence study. BMC geriatrics 2014;14(1):1.

(14) Pinkert C, Holle B. People with dementia in acute hospitals. Literature review of prevalence and reasons for hospital admission. Z Gerontol Geriatr 2012 Dec;45(8):728-734.

(15) Mukadam N, Sampson EL. A systematic review of the prevalence, associations and outcomes of dementia in older general hospital inpatients. International Psychogeriatrics 2011;23(03):344-355.

(16) Timmons S, Manning E, Barrett A, Brady NM, Browne V, O'Shea E, et al. Dementia in older people admitted to hospital: a regional multi-hospital observational study of prevalence, associations and case recognition. Age Ageing 2015 Nov;44(6):993-999.

(17) Bradshaw LE, Goldberg SE, Lewis SA, Whittamore K, Gladman JRF, Jones RG, et al. Six-month outcomes following an emergency hospital admission for older adults with co-morbid mental health problems indicate complexity of care needs. Age and Ageing 2013 September 01;42(5):582-588.

(18) Satizabal CL, Beiser AS, Chouraki V, Chêne G, Dufouil C, Seshadri S. Incidence of Dementia over Three Decades in the Framingham Heart Study. N Engl J Med 2016 02/11; 2016/02;374(6):523-532.

(19) World Health Organization. Dementia Fact sheet. April 2016; Available at: http://www.who.int/mediacentre/factsheets/fs362/en/. Accessed January 25, 2017.

(20) Fried TR, van Doorn C, O'Leary JR, Tinetti ME, Drickamer MA. Older persons' preferences for home vs hospital care in the treatment of acute illness. Arch Intern Med 2000;160(10):1501-1506.

(21) Fried TR, Bradley EH, Towle VR, Allore H. Understanding the Treatment Preferences of Seriously Ill Patients. N Engl J Med 2002 04/04; 2017/05;346(14):1061-1066.

(22) Shepperd S, Iliffe S, Doll HA, Clarke MJ, Kalra L, Wilson AD, et al. Admission avoidance hospital at home. The Cochrane Library 2016.

(23) Conley J, O'Brien CW, Leff BA, Bolen S,Zulman D. Alternative strategies to inpatient hospitalization for acute medical conditions: A systematic review. JAMA Internal Medicine 2016 November 1;176(11):1693-1702.

(24) Tibaldi V, Aimonino N, Ponzetto M, Stasi M, Amati D, Raspo S, et al. A randomized controlled trial of a home hospital intervention for frail elderly demented patients: behavioral disturbances and caregiver's stress. Arch Gerontol Geriatr 2004;38:431-436.

(25) Chan AW, Tetzlaff JM, Altman DG, Laupacis A, Gotzsche PC, Krleza-Jeric K, et al. SPIRIT 2013 statement: defining standard protocol items for clinical trials. Ann Intern Med 2013 Feb 5;158(3):200-207.

(26) Bellelli G, Morandi A, Davis DHJ, Mazzola P, Turco R, Gentile S, et al. Validation of the 4AT, a new instrument for rapid delirium screening: a study in 234 hospitalised older people. Age and Ageing 2014 March 02.

For peer review only - http://bmjopen.bmj.com/site/about/guidelines.xhtml

(27) Katzman R, Brown T, Fuld P, Peck A, Schechter R, Schimmel H. Validation of a short Orientation-Memory-Concentration Test of cognitive impairment. Am J Psychiatry 1983 Jun;140(6):734-739.
(28) Shepperd S, Harwood D, Jenkinson C, Gray A, Vessey M, Morgan P. Randomised controlled trial comparing hospital at home care with inpatient hospital care. I: three month follow up of health outcomes. BMJ 1998 Jun 13;316(7147):1786-1791.
(29) Cei M, Bartolomei C, Mumoli N. In-hospital mortality and morbidity of elderly medical patients can be predicted at admission by the Modified Early Warning Score: a prospective study. Int J Clin Pract 2009;63(4):591-595.
(30) Dundar ZD, Ergin M, Karamercan MA, Ayranci K, Colak T, Tuncar A, et al. Modified Early Warning Score and VitalPac Early Warning Score in geriatric patients admitted to emergency department. Eur J Emerg Med 2015.
(31) Ridley S. The recognition and early management of critical illness. Ann R Coll Surg Engl 2005 Sep;87(5):315-322.
(32) Aimonino Ricauda N, Tibaldi V, Leff B, Scarafiotti C, Marinello R, Zanocchi M, et al. Substitutive “hospital at home” versus inpatient care for elderly patients with exacerbations of chronic obstructive pulmonary disease: a prospective randomized, controlled trial. J Am Geriatr Soc 2008;56(3):493-500.
(33) Tibaldi V, Isaia G, Scarafiotti C, Gariglio F, Zanocchi M, Bo M, et al. Hospital at home for elderly patients with acute decompensation of chronic heart failure: a prospective randomized controlled trial. Arch Intern Med 2009;169(17):1569-1575.
(34) Worth A, Tierney AJ. Conducting research interviews with elderly people by telephone. J Adv Nurs 1993;18(7):1077-1084.
(35) Subbe CP, Kruger M, Rutherford P, Gemmel L. Validation of a modified Early Warning Score in medical admissions. QJM 2001 Oct;94(10):521-526.
(36) American College of Emergency Physicians, American Geriatrics Society, Emergency Nurses Association, Society for Academic Emergency Medicine, Geriatric Emergency Department Guidelines Task Force. Geriatric emergency department guidelines. Ann Emerg Med 2014 May;63(5):e7-25.
(37) Charlson ME, Pompei P, Ales KL, MacKenzie CR. A new method of classifying prognostic comorbidity in longitudinal studies: development and validation. J Chronic Dis 1987;40(5):373-383.
(38) Oud F, de Rooij S, Schuurman T, Duijvelaar K, van Munster B. [Predictive value of the VMS theme'Frail elderly': delirium, falling and mortality in elderly hospital patients]. Ned Tijdschr Geneeskd 2014;159:A8491-A8491.
(39) Katz S, Ford AB, Moskowitz RW, Jackson BA, Jaffe MW. Studies of illness in the aged: the index of ADL: a standardized measure of biological and psychosocial function. JAMA 1963;185(12):914-919.
(40) Herdman M, Gudex C, Lloyd A, Janssen M, Kind P, Parkin D, et al. Development and preliminary testing of the new five-level version of EQ-5D (EQ-5D-5L). Quality of Life Research 2011;20(10):1727-1736.
(41) Makai P, Brouwer WBF, Koopmanschap MA, Nieboer AP. Capabilities and quality of life in Dutch psycho-geriatric nursing homes: an exploratory study using a proxy version of the ICECAP-O. Quality of Life Research 2012;21(5):801-812.
(42) van Exel NJ, Scholte op Reimer WJ, Brouwer WB, van den Berg B, Koopmanschap MA, van den Bos GA. Instruments for assessing the burden of informal caregiving for stroke patients in clinical practice: a comparison of CSI, CRA, SCQ and self-rated burden. Clin Rehabil 2004 Mar;18(2):203-214.
(43) Robinson BC. Validation of a Caregiver Strain Index. Journal of Gerontology 1983 May 01;38(3):344-348.
(44) Bouwmans C, Hakkaart-van Roijen L, Koopmanschap M, Krol M, Severens H, Brouwer W. Handleiding iMTA Medical Cost Questionnaire (iMCQ). Rotterdam: iMTA, Erasmus Universiteit Rotterdam, 2013.
(45) Gavinski K, Carnahan R, Weckmann M. Validation of the delirium observation screening scale in a hospitalized older population. Journal of Hospital Medicine 2016:n/a-n/a.
(46) Stratton RJ, Hackston A, Longmore D, Dixon R, Price S, Stroud M, et al. Malnutrition in hospital outpatients and inpatients: prevalence, concurrent validity and ease of use of the ‘malnutrition universal screening tool’(‘MUST’) for adults. Br J Nutr 2004;92(05):799-808.
(47) Hawker GA, Mian S, Kendzerska T, French M. Measures of adult pain: Visual Analog Scale for Pain (VAS Pain), Numeric Rating Scale for Pain (NRS Pain), McGill Pain Questionnaire (MPQ), Short-Form McGill Pain Questionnaire (SF-MPQ), Chronic Pain Grade Scale (CPGS), Short Form-36 Bodily Pain Scale (SF-36 BPS), and Measure of Intermittent and Constant Osteoarthritis Pain (ICOAP). Arthritis Care & Research 2011;63(S11):S240-S252.
(48) de Haes,J.C., van Knippenberg,F.C., Neijt JP. Measuring psychological and physical distress in cancer patients: structure and application of the Rotterdam Symptom Checklist. Br J Cancer 1990 12;62(6):1034-1038.
(49) MacKnight C, Rockwood K. A Hierarchical Assessment of Balance and Mobility. Age Ageing 1995 Mar;24(2):126-130.

For peer review only - http://bmjopen.bmj.com/site/about/guidelines.xhtml

(50) Attkisson CC, Greenfield TK. The UCSF Client Satisfaction Scales: I. The Client Satisfaction Questionnaire-8. 1999.

(51) Reichheld FF. The one number you need to grow. Harv Bus Rev 2003;81(12):46-55.

(52) Quinn TJ, Dawson J, Lees JS, Chang T, Walters MR, Lees KR, et al. Time Spent at Home Poststroke: “Home-Time” a Meaningful and Robust Outcome Measure for Stroke Trials. Stroke 2008 January 01;39(1):231-233.

(53) Drummond MF, Sculpher MJ, Claxton K, Stoddart GL, Torrance GW. Methods for the economic evaluation of health care programmes. : Oxford university press; 2015.

(54) Hakkaart-van Roijen L, Van der Linden N, Bouwmans C, Kanters T, Tan S. Kostenhandleiding. Methodologie van kostenonderzoek en referentieprijzen voor economische evaluaties in de gezondheidszorg. In opdracht van Zorginstituut Nederland. Geactualiseerde versie 2015.

(55) Ritchie J, Spencer L. Qualitative data analysis for applied policy research. The qualitative researcher’s companion 2002;573(2002):305-329.

(56) Wet bescherming persoonsgegevens (2000, July 6). Available at: http://wetten.overheid.nl/BWBR0011468/2017-07-01/0/. Accessed October 25, 2017.

(57) Schulz KF, Altman DG, Moher D. CONSORT 2010 Statement: updated guidelines for reporting parallel group randomised trials. Trials 2010;11(1):32.

(58) Raad voor de leefomgeving en infrastructuur. Langer zelfstandig, een gedeelde opgave van wonen, zorg en welzijn. Advies van de Raad voor de Leefomgeving en infrastructuur over langer zelfstandig wonen. (2014, January 15) Available at: http://www.rli.nl/sites/default/files/rli-briefadvieslangerzelfstandigeengedeeldeopgavevanwonenzorgenwelzijndef23-113-30_0.pdf. Accessed October 25, 2017

(59) Caplan GA, Sulaiman NS, Mangin DA, Aimonino Ricauda N, Wilson AD, Barclay L. A meta-analysis of "hospital in the home". Med J Aust 2012 Nov 5;197(9):512-519.

(60) Porter ME. What Is Value in Health Care? N Engl J Med 2010 12/23; 2016/06;363(26):2477-2481.

(61) Bradshaw LE, Goldberg SE, Lewis SA, Whittamore K, Gladman J, Jones R, et al. Six-month outcomes following an emergency hospital admission for older adults with co-morbid mental health problems indicate complexity of care needs. Age Ageing 2013;42(5):582-588.

(62) Sayer C. “Time Spent at Home” — A Patient-Defined Outcome. NEJM Catalyst. 2016.

(63) Groff AC, Colla CH, Lee TH. Days Spent at Home — A Patient-Centered Goal and Outcome. N Engl J Med 2016 10/27; 2017/01;375(17):1610-1612.

For peer review only

For peer review only - http://bmjopen.bmj.com/site/about/guidelines.xhtml

STANDARD PROTOCOL ITEMS: RECOMMENDATIONS FOR INTERVENTIONAL TRIALS

SPIRIT 2013 Checklist: Recommended items to address in a clinical trial protocol and related documents*

Section/item	Item No	Description	Addressed on page number
**Administrative information**			
Title	1	Descriptive title identifying the study design, population, interventions, and, if applicable, trial acronym	1
Trial registration	2a	Trial identifier and registry name. If not yet registered, name of intended registry	1
	2b	All items from the World Health Organization Trial Registration Data Set	1, 3, 4, 5, 6, 8, 9, 13, 14
Protocol version	3	Date and version identifier	all
Funding	4	Sources and types of financial, material, and other support	14
Roles and responsibilities	5a	Names, affiliations, and roles of protocol contributors	14
	5b	Name and contact information for the trial sponsor	14
	5c	Role of study sponsor and funders, if any, in study design; collection, management, analysis, and interpretation of data; writing of the report; and the decision to submit the report for publication, including whether they will have ultimate authority over any of these activities	14
	5d	Composition, roles, and responsibilities of the coordinating centre, steering committee, endpoint adjudication committee, data management team, and other individuals or groups overseeing the trial, if applicable (see Item 21a for data monitoring committee)	n/a

For peer review only - http://bmjopen.bmj.com/site/about/guidelines.xhtml

**Introduction**			
Background and rationale	6a	Description of research question and justification for undertaking the trial, including summary of relevant studies (published and unpublished) examining benefits and harms for each intervention	3
	6b	Explanation for choice of comparators	3
Objectives	7	Specific objectives or hypotheses	3
Trial design	8	Description of trial design including type of trial (eg, parallel group, crossover, factorial, single group), allocation ratio, and framework (eg, superiority, equivalence, noninferiority, exploratory)	4
**Methods: Participants, interventions, and outcomes**			
Study setting	9	Description of study settings (eg, community clinic, academic hospital) and list of countries where data will be collected. Reference to where list of study sites can be obtained	4
Eligibility criteria	10	Inclusion and exclusion criteria for participants. If applicable, eligibility criteria for study centres and individuals who will perform the interventions (eg, surgeons, psychotherapists)	6
Interventions	11a	Interventions for each group with sufficient detail to allow replication, including how and when they will be administered	7, 8
	11b	Criteria for discontinuing or modifying allocated interventions for a given trial participant (eg, drug dose change in response to harms, participant request, or improving/worsening disease)	7, 8
	11c	Strategies to improve adherence to intervention protocols, and any procedures for monitoring adherence (eg, drug tablet return, laboratory tests)	n/a
	11d	Relevant concomitant care and interventions that are permitted or prohibited during the trial	9, 10
Outcomes	12	Primary, secondary, and other outcomes, including the specific measurement variable (eg, systolic blood pressure), analysis metric (eg, change from baseline, final value, time to event), method of aggregation (eg, median, proportion), and time point for each outcome. Explanation of the clinical relevance of chosen efficacy and harm outcomes is strongly recommended	9, 10
Participant timeline	13	Time schedule of enrolment, interventions (including any run-ins and washouts), assessments, and visits for participants. A schematic diagram is highly recommended (see Figure)	10

For peer review only - http://bmjopen.bmj.com/site/about/guidelines.xhtml

Sample size	14	Estimated number of participants needed to achieve study objectives and how it was determined, including clinical and statistical assumptions supporting any sample size calculations	6
Recruitment	15	Strategies for achieving adequate participant enrolment to reach target sample size	5
**Methods: Assignment of interventions (for controlled trials)**			
Allocation:			
Sequence generation	16a	Method of generating the allocation sequence (eg, computer-generated random numbers), and list of any factors for stratification. To reduce predictability of a random sequence, details of any planned restriction (eg, blocking) should be provided in a separate document that is unavailable to those who enrol participants or assign interventions	4
Allocation concealment mechanism	16b	Mechanism of implementing the allocation sequence (eg, central telephone; sequentially numbered, opaque, sealed envelopes), describing any steps to conceal the sequence until interventions are assigned	4
Implementation	16c	Who will generate the allocation sequence, who will enrol participants, and who will assign participants to interventions	4
Blinding (masking)	17a	Who will be blinded after assignment to interventions (eg, trial participants, care providers, outcome assessors, data analysts), and how	4
	17b	If blinded, circumstances under which unblinding is permissible, and procedure for revealing a participant's allocated intervention during the trial	n/a
**Methods: Data collection, management, and analysis**			
Data collection methods	18a	Plans for assessment and collection of outcome, baseline, and other trial data, including any related processes to promote data quality (eg, duplicate measurements, training of assessors) and a description of study instruments (eg, questionnaires, laboratory tests) along with their reliability and validity, if known. Reference to where data collection forms can be found, if not in the protocol	9, 10, 11
	18b	Plans to promote participant retention and complete follow-up, including list of any outcome data to be collected for participants who discontinue or deviate from intervention protocols	11

For peer review only - http://bmjopen.bmj.com/site/about/guidelines.xhtml

Data management	19	Plans for data entry, coding, security, and storage, including any related processes to promote data quality (eg, double data entry; range checks for data values). Reference to where details of data management procedures can be found, if not in the protocol	11
Statistical methods	20a	Statistical methods for analysing primary and secondary outcomes. Reference to where other details of the statistical analysis plan can be found, if not in the protocol	12
	20b	Methods for any additional analyses (eg, subgroup and adjusted analyses)	n/a
	20c	Definition of analysis population relating to protocol non-adherence (eg, as randomised analysis), and any statistical methods to handle missing data (eg, multiple imputation)	n/a
**Methods: Monitoring**			
Data monitoring	21a	Composition of data monitoring committee (DMC); summary of its role and reporting structure; statement of whether it is independent from the sponsor and competing interests; and reference to where further details about its charter can be found, if not in the protocol. Alternatively, an explanation of why a DMC is not needed	12
	21b	Description of any interim analyses and stopping guidelines, including who will have access to these interim results and make the final decision to terminate the trial	12
Harms	22	Plans for collecting, assessing, reporting, and managing solicited and spontaneously reported adverse events and other unintended effects of trial interventions or trial conduct	12
Auditing	23	Frequency and procedures for auditing trial conduct, if any, and whether the process will be independent from investigators and the sponsor	n/a
**Ethics and dissemination**			
Research ethics approval	24	Plans for seeking research ethics committee/institutional review board (REC/IRB) approval	12
Protocol amendments	25	Plans for communicating important protocol modifications (eg, changes to eligibility criteria, outcomes, analyses) to relevant parties (eg, investigators, REC/IRBs, trial participants, trial registries, journals, regulators)	12

For peer review only - http://bmjopen.bmj.com/site/about/guidelines.xhtml

Consent or assent	26a	Who will obtain informed consent or assent from potential trial participants or authorised surrogates, and how (see Item 32)	5
	26b	Additional consent provisions for collection and use of participant data and biological specimens in ancillary studies, if applicable	n/a
Confidentiality	27	How personal information about potential and enrolled participants will be collected, shared, and maintained in order to protect confidentiality before, during, and after the trial	10
Declaration of interests	28	Financial and other competing interests for principal investigators for the overall trial and each study site	13
Access to data	29	Statement of who will have access to the final trial dataset, and disclosure of contractual agreements that limit such access for investigators	10
Ancillary and post-trial care	30	Provisions, if any, for ancillary and post-trial care, and for compensation to those who suffer harm from trial participation	Available on request
Dissemination policy	31a	Plans for investigators and sponsor to communicate trial results to participants, healthcare professionals, the public, and other relevant groups (eg, via publication, reporting in results databases, or other data sharing arrangements), including any publication restrictions	12
	31b	Authorship eligibility guidelines and any intended use of professional writers	13
	31c	Plans, if any, for granting public access to the full protocol, participant-level dataset, and statistical code	n/a
**Appendices**			
Informed consent materials	32	Model consent form and other related documentation given to participants and authorised surrogates	Available on request
Biological specimens	33	Plans for collection, laboratory evaluation, and storage of biological specimens for genetic or molecular analysis in the current trial and for future use in ancillary studies, if applicable	n/a

*It is strongly recommended that this checklist be read in conjunction with the SPIRIT 2013 Explanation & Elaboration for important clarification on the items. Amendments to the protocol should be tracked and dated. The SPIRIT checklist is copyrighted by the SPIRIT Group under the Creative Commons "Attribution-NonCommercial-NoDerivs 3.0 Unported" license.

For peer review only - http://bmjopen.bmj.com/site/about/guidelines.xhtml

BMJ Open

Hospital at Home care for older patients with cognitive impairment: A protocol for a randomized controlled feasibility trial

Journal:	*BMJ Open*
Manuscript ID	bmjopen-2017-020332.R1
Article Type:	Protocol
Date Submitted by the Author:	21-Dec-2017
Complete List of Authors:	Pouw, Maaike; University of Groningen, University Medical Center Groningen, Department of Geriatrics; Martini Hospital, Department of Internal Medicine<br>Calf, Agneta; University of Groningen, University Medical Center Groningen, Department of Geriatrics<br>van Munster, Barbara; University of Groningen, University Medical Center Groningen, Department of Geriatrics; Gelre Hospitals, Department of Geriatrics<br>ter Maaten, Jan; University of Groningen, University Medical Center Groningen, Department of Internal Medicine, Emergency Department<br>Smidt, Nynke; University of Groningen, University Medical Center Groningen, Department of Epidemiology, The Netherlands<br>De Roolj, Sophia; University of Groningen, University Medical Center Groningen, Department of Geriatrics
**Primary Subject Heading**:	Geriatric medicine
Secondary Subject Heading:	Patient-centred medicine
Keywords:	Dementia < NEUROLOGY, Organisation of health services < HEALTH SERVICES ADMINISTRATION & MANAGEMENT, GERIATRIC MEDICINE, Delirium & cognitive disorders < PSYCHIATRY, INTERNAL MEDICINE

SCHOLARONE™
Manuscripts

Public title

Hospital at Home care for older patients with cognitive impairment and an acute medical illness

Scientific title

Hospital at Home care for older patients with cognitive impairment: A protocol for a randomized controlled feasibility trial

Maaike A Pouw,[1,2] Agneta H Calf,[1] Barbara C van Munster,[1,3] Jan C ter Maaten,[4] Nynke Smidt,[1,5] & Sophia E de Rooij [1]

Acronym

H@H

Abstract

INTRODUCTION: An acute hospital admission is a stressful life event for older people, particularly for those with cognitive impairment. The hospitalisation is often complicated by hospital-associated geriatric syndromes, including delirium and functional loss, leading to functional decline and nursing home admission. Hospital at Home care aims to avoid hospitalisation-associated adverse outcomes in older patients with cognitive impairment by providing hospital care in the patient's own environment.

METHODS AND ANALYSIS: This randomized, non-blinded feasibility trial aims to assess the feasibility of conducting a randomized controlled trial in terms of the recruitment, use and acceptability of Hospital at Home care for older patients with cognitive impairment. The quality of care will be evaluated and the advantages and disadvantages of the Hospital at Home care program compared to usual hospital care. Eligible patients will be randomized either to Hospital at Home care in their own environment or usual hospital care. The intervention consists of hospital level care provided at patients' homes, including visits from health care professionals, diagnostics (laboratory tests, blood cultures) and treatment. The control group will receive usual hospital care. Measurements will be conducted at baseline, during admission, at discharge and at three and six months after the baseline assessment.

ETHICS AND DISSEMINATION: Institutional ethics approval has been granted. The findings will be disseminated through public lectures, professional and scientific conferences, as well as peer-reviewed journal articles. The study findings will contribute to knowledge on the implementation of Hospital at Home care for older patients with cognitive disorders. The results will be used to inform and support strategies to deliver eligible care to older patients with cognitive impairment.

TRIAL REGISTRATION: This study was registered with the NTR registry (NTR6581) on 26 July 2017.

Keywords: Dementia, Cognition disorders, Central Nervous System Diseases, Aged, Home Care Services; Hospital-Based, Hospitalization, Randomized Controlled Trials, Hospital at Home

For peer review only - http://bmjopen.bmj.com/site/about/guidelines.xhtml

Strengths and limitations of this study

- This study addresses the feasibility of Hospital at Home care in patients with cognitive impairment, a patient population that is often excluded from participation in scientific research
- A process evaluation facilitates the investigation of factors that influence the experiences and perceptions of all persons involved in Hospital at Home care
- Stakeholders were involved in the development of the design of the study which will support the implementation of Hospital at Home care and a future trial
- Because of a limited sample size due to the study being centered on feasibility, results will not show effectiveness of Hospital at Home care compared to usual hospital care.

Introduction

Background

An acute hospital admission is a stressful life event, particularly for older people. In addition to the stress of an acute illness, the hospital admission itself contributes to this stress.[1] Older hospitalized patients are often deprived of sleep, and they spend an average of 20 of every 24 hours in bed, they become poorly nourished, and experience sensory deprivation or overstimulation, resulting in confusion.[2-5] These adverse effects of hospitalization contribute to the occurrence of geriatric conditions, such as delirium, functional decline, falls, incontinence, hospital acquired infections and pressure injuries.[6-9] Adverse effects of hospitalization occur more easily in older people, particularly in those who are already frail, a growing portion of the worldwide ageing population.[10,11] Frailty is a state of increased vulnerability to external stressors resulting from aging-associated declines in reserve and function across multiple physiologic systems.[10] Cognitive impairment (i.e., dementia) is an important contributor to frailty in older people.[12] Cognitively impaired older people are more likely to become hospitalised and once admitted, they experience longer stays than their peers without cognitive impairment.[13-15] The combination of hospitalization and cognitive impairment in older people is associated with further functional and cognitive declines and higher mortality rates, and it leads to more discharges to long-term care facilities.[16,17] The prevalence and worldwide burden of cognitive impairment will continue to increase as the average life expectancy increases.[18] The total number of people with cognitive impairment is estimated to be 75.6 million in 2030 and will nearly triple in 2050 to 135.5 million.[19] An increase in the number of hospital admissions of older people with cognitive impairment and an increase in number of hospitalisation-associated adverse outcomes are therefore to be expected.

For peer review only - http://bmjopen.bmj.com/site/about/guidelines.xhtml

Besides adverse outcomes of hospitalization, many older people and their caregivers do not necessarily desire a hospital admission in case of an acute illness or exacerbation of a chronic illness. Fried et al. (2000) have studied the preferences of community-dwelling persons 65 years of age and older who were hospitalized with a primary diagnosis of congestive heart failure, chronic obstructive pulmonary disease or pneumonia. The authors reported that over 50% of older patients preferred to receive hospital treatment at home, because they felt that their homes were more comfortable.[20] In the treatment preferences of seriously ill patients 60 years of age and older, the likelihood of cognitive and functional impairment as an adverse outcome of the treatment was weighed in the decision-making process. There was a substantial decrease in the number of participants who opted for treatment if the likelihood of impairment after treatment was 50% or higher.[21]

Hospital at Home care could provide an effective alternative to inpatient care for a select group of elderly patients now requiring hospitalisation. Hospital at Home care is coordinated, multidisciplinary care in the homes of people who would otherwise be admitted to the hospital. Hospital at Home care is an accepted alternative to inpatient hospital level care in several countries (e.g., the United States, Australia, Italy and the United Kingdom) but not yet in the Netherlands.[22] Since the 1990s, Hospital at Home has been evaluated in (older) persons with various acute medical conditions, such as heart failure, exacerbations in chronic obstructive pulmonary disease (COPD) and infections (e.g., cellulitis, pneumonia).[22] In systematic reviews comparing alternative strategies to inpatient hospitalization, lower or equal mortality rates and return hospitalization rates (i.e., subsequent admissions after discharge) were found for Hospital at Home care, there was a lower incidence of delirium, and there was a positive effect on patient and caregiver satisfaction.[22-24] Only one completed trial conducted in Italy included 109 patients with cognitive impairment (i.e. dementia), Tibaldi et al. reported a positive effect of a Hospital at Home intervention on behavioural disturbances and caregiver stress in patients with dementia.[25] Results of a still on-going trial including people with i.a. cognitive impairment in the United Kingdom, will follow in the near future.[26] Whether Hospital at Home care provides a suitable alternative with regard to other outcomes as patient satisfaction, quality of care, hospitalisation-associated adverse events and costs in older people with cognitive impairment remains unclear and further research is needed. Therefore, our primary aim is to investigate the feasibility of a Hospital at Home care program for older patients with cognitive impairment in terms of the patient recruitment, use and acceptability, and secondly to investigate the advantages and disadvantages of Hospital at Home care compared to usual hospital care from different perspectives.

Objectives of this study are:

For peer review only - http://bmjopen.bmj.com/site/about/guidelines.xhtml

1) To assess the participation rate of the Hospital at Home trial among patients 65 years and older with cognitive impairment, acute illness, and emergency hospital admission. What are the reasons for non-participation?

2) To assess the potential advantages and disadvantages of Hospital at Home care and usual hospital care for the patients, caregivers and Dutch medical health system.

3) To assess the feasibility of Hospital at Home care in terms of the quality of care with regard to geriatric syndromes, institutionalization, mortality, total days with urinary catheter, length of stay (in the hospital or in Hospital at Home care) and timing/intensity of the contact with health care professionals.

Methods and analysis

Trial design

The design is a randomized controlled feasibility trial and will utilize a process evaluation. This study will be conducted at the medical emergency department (ED) of the academic hospital of the University Medical Center of Groningen in the Netherlands and will evaluate cognitively impaired older patients who are in need of acute hospital care. Figure 1 shows the trial design summary. Participants will be randomized to either Hospital at Home care or usual hospital care in a 4:1 ratio, respectively. Patients will be randomized using a computerized random number generator (http://www.randomization.com), including block randomization.

An independent research nurse who is not involved in the patient care will complete the baseline assessment and allocate the participants (using sealed sequenced envelopes) into the Hospital at Home care (intervention) or usual hospital care group (control). The research nurse will not be aware of the randomization method. The participants, health care professionals and research staff will not be blinded to the intervention. The reporting of the design of this trial protocol is in accordance with the SPIRIT 2013 statement for clinical trial protocols.[27]

For peer review only - http://bmjopen.bmj.com/site/about/guidelines.xhtml

Figure 1

Study population

Patients 65 years of age and older who are admitted to the medical emergency department (ED) will be identified by the ED staff as potential eligible patients. Subsequently, the ED staff will inform the research nurse. The research nurse will complete the eligibility criteria checklist (table 1). The research nurse will ask the patient and their informal caregiver (i.e., partner, child, relative, friend) for their willingness to participate in the study and to provide written informed consent. The patient and informal caregiver will need to both sign the informed consent form before the patient can participate in the H@H trial.

An evaluation to assess the mental capacity is conducted by the involved ED staff in the setting of the emergency department assessment. If the participant lacks the capacity to consent (mentally incapacitated), and an informal caregiver (i.e., partner, child, friend) is present, this informal caregiver will be asked to act as a personal consultee. The personal consultee will determine whether he or she believes that participation in the study would be in accordance with the values and interests of the individual and will subsequently sign the patient's informed consent form.

For peer review only - http://bmjopen.bmj.com/site/about/guidelines.xhtml

Table 1 Patient eligibility criteria for participation in Hospital at Home trial

Inclusion criteria	Exclusion criteria
▪ Age 65 years of age and older<br>▪ Cognitive impairment, i.e. dementia, delirium or other cause of cognitive impairment, and either:<br>○ previously diagnosed or documented in the medical records or<br>○ identified by the ED-clinician (e.g., with the 4AT-test and/or Six item cognitive impairment test)[28-30]<br>▪ Presented at the ED with a defined acute illness<br>▪ Required hospital admission, according to the attending ED physician but not expected to require emergency interventions<br>○ Modified Early Warning Score (MEWS) ≤ 2 points[31-33]<br>▪ Living in hospital's catchment area (< 25 km)<br>▪ Informal caregiver is present and able to understand and perform instructions and consented to participate in the trial<br>▪ Home suitable for Hospital at Home care (available informal caregiver, running water, adequate heating, safety) [30]	▪ Previously enrolled<br>▪ Hospitalized within the seven days preceding ED presentation<br>▪ Nursing home residents or awaiting a nursing home place on an active waiting list (excluding so-called sleeping waiting list candidates)[27]<br>▪ Additional care needed<br>○ Required surgical assessment<br>○ Suspected acute coronary syndrome or cardiac arrythmia[31]<br>○ Dialysis dependent patients[31]<br>○ Expected terminal events[31] or in need of diagnostic or palliative care due to oncological or haematological illness

ED= Emergency department

Sample size of study population

Based on the numbers available from the emergency department (ED) of the University Medical Center Groningen, the Netherlands, we calculated that an average of 3990 older patients 65 years and over is admitted to our medical ED each year. The Hospital at Home trial will be introduced during working hours, which provides an estimated 1900 patients per year. Not all 1900 patients will be eligible for study inclusion. Based on screening of ED medical records, approximately 15% of the patients meet the eligibility criteria for Hospital at Home care, resulting in 285 eligible persons per year. In recent randomized controlled trials (RCTs) of Hospital at Home care in Italy, 54% and 57% of the eligible patients was willing to participate and gave informed consent.[34,35] We presume a similar consent rate of 50%, as described in these previous clinical trials, and expect for 143 patients to be included.

Study procedures

After (written) informed consent is obtained, all participants will complete two brief tests to assess cognitive impairment, and the participant and caregiver will complete the baseline assessment. Subsequently, randomization takes place to either (a) the Hospital at Home care-intervention, translocation of care from the hospital to a participants' home or (b) the control group, usual hospital care. All care will be delivered according to hospital protocols, current regulations and

For peer review only - http://bmjopen.bmj.com/site/about/guidelines.xhtml

guidelines and, if needed, described in the standard operating procedures (SOPs). If no informed consent is given by either the patient or the caregiver, the reasons for non-participation, date of birth, sex of the patient and the relationship between patient and caregiver will be reported.

Intervention

Hospital at Home care

Hospital at Home care will be delivered by a multidisciplinary team consisting of a physician, nurse, pharmacist and physiotherapist. Depending on the participants' needs, other disciplines (e.g., a dietician, occupational therapist or social services) can be involved in the Hospital at Home care. The day-to-day care will be provided by the nurse and physician visiting the participant. The Hospital at Home care team works under the responsibility of the medical specialist in the hospital, and 24/7 consultation of the expertise and services of the hospital is part of the protocol.

The participants allocated to Hospital at Home care will receive hospital level care in their own homes. After a stay of one night in the hospital, while the Hospital at Home care arrangements are being made, the participant will be transferred home and receive Hospital at Home care. The Hospital at Home nurse is responsible for the day-to-day care and will be present upon arrival of the participant at his/her residence. Hospital at Home care is described in the care protocols including SOPs and could include intravenous therapy (e.g., antibiotics, fluid, and/or diuretics), oxygen therapy, and/or nebulizer, indwelling urine catheter or a nasopharyngeal food tube. After the care intake and a period of direct nursing supervision, the participant will receive intermittent nursing visits daily (starting with 3x/day), including weekends and public holidays. The Hospital at Home physician will make a home visit every day (excl. weekends). The Hospital at Home physician and nurse will be available for emergency visits. The participant will receive a medical alert device in the house, with a 24/7 connection to an on-call service. Alert instructions will be explained to the participant and caregiver. A physiotherapist will visit the participant at home to evaluate any problems with balance and/or walking and immobility. The Hospital at Home team works under the supervision of the hospital medical specialist. Daily screenings and measurements will be recorded in a Hospital at Home record, which stays with the participant. Diagnostic procedures and therapeutics that cannot wait and are not available at home, such as endoscopy or CT-scan, will be arranged through brief visits to the hospital. The participant will be 'admitted' to the Hospital at Home care for as long as indicated.

Discharge from Hospital at Home

If the participant recovers to such an extent that hospital level care is no longer needed, the participant will be discharged from the Hospital at Home care program, similar to the discharge

For peer review only - http://bmjopen.bmj.com/site/about/guidelines.xhtml

procedure when the participant would receive usual hospital care. Hospital at Home care will end with discharge planning with the participant, family, Hospital at Home physician and nurse. The discharge plan includes follow-up appointments (e.g., at the hospital or general practitioner), information on medication, warning signs and symptoms and an on-going management plan. All hospital-related care equipment will be removed from the participants' house, and arrangements with homecare agencies and/or paramedical staff will be reviewed and adjusted to the current situation.

Hospital care as usual

Participants allocated to the control arm will be admitted to a hospital ward and receive usual hospital care. After admission and intake on the ward, the participant will receive intermittent visits from the ward nurse multiple times a day. The ward physician will visit once every day (excl. weekends), with extra visits provided if needed. An emergency alert device, through which nurses and physician can be contacted, will be placed next to the bed. A physiotherapist will visit the participant at the ward to address problems with balance and/or walking and immobility. Depending on the participants' needs, other disciplines (e.g., a dietician, occupational therapist or social services) can be involved in the hospital care. The medical record is the hospital record and additional H@H research forms will be added to this record for research purposes. The participant will be admitted to the hospital for as long as indicated.

Hospital discharge

If the participant recovers to such an extent that hospital level care is no longer needed, the participant will be discharged from the hospital after discharge planning with the participant, family, physician and nurse. The discharge plan includes follow-up appointments (e.g., at the hospital or general practitioner), information on medication, warning signs and symptoms and an on-going management plan. Arrangements with homecare agencies and/or paramedical staff will be reviewed and adjusted to the current situation.

Follow-up

At three and six months following randomization, all participants will be contacted for an interview by telephone[36] or a face-to-face interview, if needed. The participants will be allowed to receive support with these questions from their relatives or informal caregivers. An interview will require a maximum of 30 minutes. In case of institutionalization or mortality, this event will be recorded. Additionally, information on hospital readmission and length of stay will be collected from the

For peer review only - http://bmjopen.bmj.com/site/about/guidelines.xhtml

hospital administration system and health insurers. Mortality and nursing home placements will be collected from registries from the general practitioner and municipalities.

Timing of measurements and outcome measures

Data will be collected at baseline at the emergency department, during admission (in Hospital at Home or hospital), at discharge and at three and six months following randomization, plus or minus two weeks. An overview of the timing of measurements and outcome measures are shown in Table 2.

Feasibility

For the participation rates, the proportion of participants per step will be calculated. The reasons for non-participation and data concerning the characteristics of non-participants will be collected. We consider the participation rate feasible when it is similar to the participation rate as is described in previous RCTs and around 50% of the eligible patients will consent to participate.[34,35] Quality of care will be measured by collecting data on patient, institutionalization (e.g., to the hospital or nursing home), mortality, ADL-functioning, prevalence of hospitalisation-associated geriatric syndromes, the length of stay in the hospital or Hospital at Home care program and contact with health care professionals. The study is considered feasible if the quality of care of Hospital at Home care on these measurements is non- inferior to usual hospital care.

For peer review only - http://bmjopen.bmj.com/site/about/guidelines.xhtml

Table 2 Overview of the content and description of outcome measures and timing of measurements

	**Description and instrument**	Timing of measurements					
		screening	baseline	admission	discharge	3 months	6 months
Degree of illness based on physiological parameters	Vital signs alarm score; Modified Early Warning Score, [37]	R					
Cognitive impairment	4AT-test for delirium* Six item cognitive impairment test for cognitive impairment*,[28,38]	R					
Socio-demographics	Date of birth, nationality, household composition, marital status, highest level of education		R				
Health status	Charlson co-morbidity index*,[39]		R				
Identifying at-risk patients	Safety management system patient screening (VMS),[40]		R				
Functional status	Activities of daily living, modified Katz-ADL index score,[41]		R		R	R	R
Health status	EQ-5d-5l*,[42]		R		R	R	R
(Health-related) quality of life, well-being	Icepop capability measure for Older people (ICECAP-O)*[43]		R		R	R	R
Caregiver burden	Self-rated burden scale*, caregiver strain index*,[44,45]		C		C	C	C
Medical consumption	Imta Medical Consumption Questionnaire (imcq)*,[46]		P,C			P,C	P,C
Hospitalisation-associated geriatric syndromes	Infections, falls, pressure injuries, in case of delirium; delirium observation scale score (DOSS)[47] and use of physical or chemical restraints, total days with a urinary catheter			N			
Nutrition	Malnutrition Universal Screening Tool (MUST),[48]		N				
	Food intake, fluid intake			N			
Pain	Numeric Rating Scale-score (NRS) for pain [49]			N			
Health perception	(Rotterdam) symptom checklist*[50]			N			
Immobility	Hierarchical assessment of balance and mobility (HABAM)[51]			Ph			
Satisfaction with care	Client Satisfaction Questionnaire 8 (CSQ-8)*, care evaluation question*,[52,53]				P<br>P,C,<br>N,D		
Mortality	Mortality at 30 days, 3 months and 6 months after baseline*				N	R	R
(Re)admission hospital	Length of stay, readmission rate at 30 days, 3 months and 6 months after baseline*				N	R	R

Assessed by: P= Participant, C=Caregiver, N= Nurse, D= Doctor, R= Research nurse, Ph=Physiotherapist.
(*)= All assessments marked by an asterisk are extra for trial purposes and are not part of the medical treatment

Other outcomes measures

Advantages and disadvantages of the Hospital at Home care program will be assessed through multiple instruments and questionnaires. The instruments are validated and used in community-dwelling older patients with cognitive disorders. Additional data will be collected on the time spent at home (home-time); total number of days alive and out of the hospital or a skilled nursing facility in the 6 months following the randomization at the emergency department,[54] the number of transfers (home ↔ hospital) and the number of health care professionals involved.

For peer review only

For peer review only - http://bmjopen.bmj.com/site/about/guidelines.xhtml

Cost data will be collected, as described by Drummond et al., including the costs to the health care system, patients and families, and other sectors.[55] The volume of care use will be extracted from hospital files and combined with the reference cost values, as provided by the cost guidance module of the Dutch National Health Care Institute.[56]

Process evaluation

A process evaluation will be conducted as part of the feasibility study to understand the barriers and facilitators to participate and to gain an understanding of the experiences and perceptions of Hospital at Home care of participants and health care professionals. From all eligible patients who declined to participate, data concerning the patient characteristics and reasons for non-participation will be collected. At the end of the trial, a representative sample of participants and/or their informal caregivers will be invited for an interview to evaluate their experiences receiving Hospital at Home care. The interviewer will not be a an active member of the research team or involved in day-to-day care and will explore independently how the participants perceived Hospital at Home care, including the contact with the health professionals and the impact of Hospital at Home care on their lives and their caregivers' personal lives. In case of participant dropout, efforts will be made to obtain an understanding of why the participants did not complete the trial.

In addition, a representative sample of health care professionals, consisting of physicians and nurses working in the Emergency Department, physicians and nurses providing the Hospital at Home care, and general practitioners, will be asked to participate in a face-to-face interview. The health care professionals will be asked about their experiences and opinions about the H@H-trial and Hospital at Home care. All interviews will be transcribed verbatim, and a framework analysis will be used as the method of qualitative data analysis.[57]

Data management

All data will be entered in an electronic trial-specific database, with the participants identified by a unique trial number. Confidentiality of participant information will be maintained throughout the trial. Information can only be traced to the participants by designated researchers. The database will be stored and maintained by Castor Electronic Data Capture, compliant with GCP guidelines and the European Data Protection Directive (Castor Electronic Data Capture, Ciwit BV, Amsterdam, the Netherlands, 2017). Data will be stored for a maximum period of 15 years after the study has ended, according to Dutch law.[58]

For peer review only - http://bmjopen.bmj.com/site/about/guidelines.xhtml

Statistical analysis

The participant flow diagram, according to CONSORT guidelines,[59] will provide a summary of the recruitment and declination rates in percentage (%) at baseline, discharge and three- and six-month follow-ups. Distributions of the data at baseline, discharge and three and six months after randomization will be explored, with unusual values noted and explained. Variables will be summarized as the n (%), mean (standard deviation) or median (interquartile range) for each group, to characterize the sample and search for any imbalances. The percentages, means and standard deviation, and medians and interquartile ranges will be calculated to describe the quality of care and the advantages and disadvantages of Hospital at Home care at baseline, discharge and three- and six-month follow-ups.

Monitoring and participant safety

Although, the H@H trial is considered to be a low risk trial, the participant safety will be monitored by an independent Data Monitoring Committee (DMC). The DMC will consist of two members: an experienced clinician and an epidemiologist. Members of the DMC are independent of the trial and will discuss each individual participant with serious adverse events. The DMC will receive and review the serious adverse events and evaluate the risk involved with negative outcomes. The DMC is authorized to make recommendations to temporary put on hold or ending the study prematurely when participant safety is an issue, based on their findings. All serious adverse events will be reported to the principal investigator within 24 hours of knowledge of the event and then subsequently reported to the Dutch portal for medical research involving human subjects.

Discussion

Reducing unwanted hospital admissions in older patients with cognitive impairments and facilitating patient-centred care in a patient's preferred location is a goal worthy of pursuing. This goal aligns with the tenet of the current Dutch government and the advice provided by the Dutch Council for the Environment and Infrastructure: to actively promote and enable people to live independently in their own homes for as long as they desire.[60] Previous trials and a recent review have confirmed that alternative management strategies for low-risk patients with acute medical conditions conventionally treated through hospitalization exist with positive impact on patient satisfaction, are effective and can be safely achieved in lower cost settings.[22,23,61]

For peer review only - http://bmjopen.bmj.com/site/about/guidelines.xhtml

Introducing a Hospital at Home care trajectory in the Netherlands is incited by the principles of value-based health care: improving the patients experience of care and as a result of this process reducing the costs.[62] All countries with an ageing population experience pressure, in terms of shortage of (emergency) hospital beds and rising healthcare costs. Hospital at Home care could be shown beneficial in facilitating higher valued care for patients and their caregivers without additional costs. Benefit should be measured in other outcomes than clinical indicators such as mortality. To illustrate, one of the outcomes of a future RCT could be the time spent at home. Time spent at home has been defined as the total number of days alive and out of the hospital or a skilled nursing facility in the 6 months after hospital admission.[54] It has been used as a primary outcome in a follow-up study of older patients with acute hospital admissions, and has been demonstrated to be of more importance in older patients.[63-65] Evaluation of time spent a home in this feasibility study could support estimating a sample size based on a patient-relevant outcome in a future RCT.

This study will be the first to investigate the feasibility of providing acute hospital care at home for older patients with cognitive impairment in the Netherlands. Studying Hospital at Home care and identifying the barriers and facilitators will support the implementation of Hospital at Home care and break new ground for a future RCT investigating the (cost-)effectiveness.

Ethics and Dissemination

This study was approved by the Medical Research Ethics Committee of the University Medical Center Groningen in April 2017, reference number: 2016.686 The trial will be conducted in accordance with the Declaration of Helsinki 1996, principles of good clinical practice and the University Medical Center of Groningen Research Code. Any protocol amendments will be submitted to the ethics committee. A register of the protocol amendments will be available in the study protocol.

The results of the trial will be reported according to the CONSORT guidelines and will contribute to knowledge of the implementation of Hospital at Home care and patient-centred acute care for older patients with cognitive impairment. The study will also contribute to the knowledge of the transmural cooperation and costs of providing care, in terms of the translocation of hospital care to home. Regularly updates will be published on the study website and in newsletters. Conferences and meetings will be held for all involved health care professionals. Participants who requested information on the study will be sent a lay summary. A publication policy will be agreed upon with co-applicants. The study findings will be published in relevant peer-reviewed journals.

For peer review only - http://bmjopen.bmj.com/site/about/guidelines.xhtml

Authors' affiliations
[1] Department of Geriatrics, University Medical Center Groningen, University of Groningen, Groningen, the Netherlands
[2] Department of Internal Medicine, Martini Hospital, Groningen, The Netherlands.
[3] Department of Geriatrics, Gelre Hospitals, Apeldoorn, The Netherlands.
[4] Department of Internal Medicine, Emergency Department, University Medical Center Groningen, University of Groningen, Groningen, The Netherlands
[5] Department of Epidemiology, University of Groningen, University Medical Center Groningen, Groningen, The Netherlands.

Acknowledgements The authors would like to thank all care professionals of Alzheimer Nederland, Espria, Icare, TSN, Buurtzorg, umcGroningen thuis, MCZ, Netwerk Dementie Groningen, Netwerk Dementie Drenthe, Gemeente Groningen, WIJ Groningen, Zorgbelang, Healthy Aging Network Northern Netherlands, Stichting Effectieve Ouderenzorg, ZonMW, Deltaplan Dementie, Nationaal Programma Ouderenzorg, Zorg innovatie Forum and the elderly of Denktank 60+ Noord for their time, critical appraisal and advise.

Contributors MP drafted the manuscript. All authors were involved in making substantial contributions to the conception and design of the protocol and the critical revision of the manuscript for publication. All authors approved the final version to be published.

Funding This study is independent research funded by ZonMw (project Memorabel, Deltaplan Dementie), project number #733050401 (contact: memorabel@zonmw.nl). Additional funding is provided by the Dutch Ministry of Health, Welfare and Sport (Ministerie van Volksgezondheid, Welzijn en Sport) and the University Medical Center Groningen.

Disclaimer The views and opinions expressed in this paper are those of the authors and not necessarily those of the UMCG, Memorabel, Deltaplan Dementie or ZonMW.

Competing interests All authors have declared that they have no competing interests

Ethics approval for this study has been obtained from the Medical Research Ethics Committee of the University Medical Center Groningen April 2017 (ref: 2016.686).

Recruitment status is pending; participants are not yet being recruited or enrolled. Anticipated date of enrolment of the first participant is December 2017.

Corresponding author: m.a.pouw@umcg.nl. University Center of Geriatric Medicine, University Medical Center Groningen. Hanzeplein 1, Postbus 30 001, 9700 RB Groningen

Scientific inquiries: prof Sophia E.de Rooij, s.e.j.a.de.rooij@umcg.nl, telephone number 050-3613921, Hanzeplein 1, Postbus 30 001, 9700 RB Groningen. University of Groningen, University Medical Center Groningen, University Center for Geriatric Medicine, Groningen, The Netherlands.
Generic email address hospitalathome@umcg.nl.

Abbreviations

ADL: Activities of Daily Living
CT: Computer Tomography
DMC: Data Monitoring Committee
ED: Emergency Department
H@H: Hospital at Home
RCT: Randomized Controlled Trial
UMCG: University Medical Center Groningen

References

For peer review only - http://bmjopen.bmj.com/site/about/guidelines.xhtml

(1) Krumholz HM. Post-Hospital Syndrome - A Condition of Generalized Risk. N Engl J Med 2013 01/10;368(2):100-102.
(2) Missildine K, Bergstrom N, Meininger J, Richards K, Foreman MD. Sleep in Hospitalized Elders: A Pilot Study. Geriatr Nurs 2010 0;31(4):263-271.
(3) Brown CJ, Redden DT, Flood KL, Allman RM. The underrecognized epidemic of low mobility during hospitalization of older adults. J Am Geriatr Soc 2009 Sep;57(9):1660-1665.
(4) Sullivan DH, Sun S, Walls RC. Protein-energy undernutrition among elderly hospitalized patients: a prospective study. JAMA 1999 Jun 2;281(21):2013-2019.
(5) Creditor MC. Hazards of hospitalization of the elderly. Ann Intern Med 1993;118(3):219-223.
(6) Inouye SK, Studenski S, Tinetti ME, Kuchel GA. Geriatric Syndromes: Clinical, Research, and Policy Implications of a Core Geriatric Concept. J Am Geriatr Soc 2007;55(5):780-791.
(7) Mudge AM, Banks MD, Barnett AG, Blackberry I, Graves N, Green T, et al. CHERISH (collaboration for hospitalised elders reducing the impact of stays in hospital): protocol for a multi-site improvement program to reduce geriatric syndromes in older inpatients. BMC Geriatrics 2017;17(1):11.
(8) Covinsky KE, Pierluissi E, Johnston CB. Hospitalization-associated disability: "She was probably able to ambulate, but I'm not sure". JAMA 2011 Oct 26;306(16):1782-1793.
(9) Zisberg A, Shadmi E, Gur-Yaish N, Tonkikh O, Sinoff G. Hospital-associated functional decline: the role of hospitalization processes beyond individual risk factors. J Am Geriatr Soc 2015 Jan;63(1):55-62.
(10) Fried LP, Tangen CM, Walston J, Newman AB, Hirsch C, Gottdiener J, et al. Frailty in older adults: evidence for a phenotype. J Gerontol A Biol Sci Med Sci 2001 Mar;56(3):M146-56.
(11) Clegg A, Young J, Iliffe S, Rikkert MO, Rockwood K. Frailty in elderly people. The Lancet 2013 3/2–8;381(9868):752-762.
(12) Ferrucci L, Guralnik JM, Studenski S, Fried LP, Cutler GB, Walston JD, et al. Designing Randomized, Controlled Trials Aimed at Preventing or Delaying Functional Decline and Disability in Frail, Older Persons: A Consensus Report. J Am Geriatr Soc 2004;52(4):625-634.
(13) Glover A, Bradshaw LE, Watson N, Laithwaite E, Goldberg SE, Whittamore KH, et al. Diagnoses, problems and healthcare interventions amongst older people with an unscheduled hospital admission who have concurrent mental health problems: a prevalence study. BMC geriatrics 2014;14(1):1.
(14) Pinkert C, Holle B. People with dementia in acute hospitals. Literature review of prevalence and reasons for hospital admission. Z Gerontol Geriatr 2012 Dec;45(8):728-734.
(15) Mukadam N, Sampson EL. A systematic review of the prevalence, associations and outcomes of dementia in older general hospital inpatients. International Psychogeriatrics 2011;23(03):344-355.
(16) Timmons S, Manning E, Barrett A, Brady NM, Browne V, O'Shea E, et al. Dementia in older people admitted to hospital: a regional multi-hospital observational study of prevalence, associations and case recognition. Age Ageing 2015 Nov;44(6):993-999.
(17) Bradshaw LE, Goldberg SE, Lewis SA, Whittamore K, Gladman JRF, Jones RG, et al. Six-month outcomes following an emergency hospital admission for older adults with co-morbid mental health problems indicate complexity of care needs. Age and Ageing 2013 September 01;42(5):582-588.
(18) Satizabal CL, Beiser AS, Chouraki V, Chêne G, Dufouil C, Seshadri S. Incidence of Dementia over Three Decades in the Framingham Heart Study. N Engl J Med 2016 02/11; 2016/02;374(6):523-532.
(19) World Health Organization. Dementia Fact sheet. April 2016; Available at: http://www.who.int/mediacentre/factsheets/fs362/en/. Accessed January 25, 2017.
(20) Fried TR, van Doorn C, O'Leary JR, Tinetti ME, Drickamer MA. Older persons' preferences for home vs hospital care in the treatment of acute illness. Arch Intern Med 2000;160(10):1501-1506.
(21) Fried TR, Bradley EH, Towle VR, Allore H. Understanding the Treatment Preferences of Seriously Ill Patients. N Engl J Med 2002 04/04; 2017/05;346(14):1061-1066.
(22) Shepperd S, Iliffe S, Doll HA, Clarke MJ, Kalra L, Wilson AD, et al. Admission avoidance hospital at home. The Cochrane Library 2016.
(23) Conley J, O'Brien CW, Leff BA, Bolen S,Zulman D. Alternative strategies to inpatient hospitalization for acute medical conditions: A systematic review. JAMA Internal Medicine 2016 November 1;176(11):1693-1702.
(24) Caplan G. Does 'Hospital in the Home' treatment prevent delirium? Aging Health 2008 02/01; 2017/12;4(1):69-74.
(25) Tibaldi V, Aimonino N, Ponzetto M, Stasi M, Amati D, Raspo S, et al. A randomized controlled trial of a home hospital intervention for frail elderly demented patients: behavioral disturbances and caregiver's stress. Arch Gerontol Geriatr 2004;38:431-436.

For peer review only - http://bmjopen.bmj.com/site/about/guidelines.xhtml

(26) Shepperd S, Cradduck-Bamford A, Butler C, Ellis G, Godfrey M, Gray A, et al. A multi-centre randomised trial to compare the effectiveness of geriatrician-led admission avoidance hospital at home versus inpatient admission. Trials 2017;18:10.1186/s13063-017-2214-y.
(27) Chan AW, Tetzlaff JM, Altman DG, Laupacis A, Gotzsche PC, Krleza-Jeric K, et al. SPIRIT 2013 statement: defining standard protocol items for clinical trials. Ann Intern Med 2013 Feb 5;158(3):200-207.
(28) Bellelli G, Morandi A, Davis DHJ, Mazzola P, Turco R, Gentile S, et al. Validation of the 4AT, a new instrument for rapid delirium screening: a study in 234 hospitalised older people. Age and Ageing 2014 March 02.
(29) Katzman R, Brown T, Fuld P, Peck A, Schechter R, Schimmel H. Validation of a short Orientation-Memory-Concentration Test of cognitive impairment. Am J Psychiatry 1983 Jun;140(6):734-739.
(30) Shepperd S, Harwood D, Jenkinson C, Gray A, Vessey M, Morgan P. Randomised controlled trial comparing hospital at home care with inpatient hospital care. I: three month follow up of health outcomes. BMJ 1998 Jun 13;316(7147):1786-1791.
(31) Cei M, Bartolomei C, Mumoli N. In-hospital mortality and morbidity of elderly medical patients can be predicted at admission by the Modified Early Warning Score: a prospective study. Int J Clin Pract 2009;63(4):591-595.
(32) Dundar ZD, Ergin M, Karamercan MA, Ayranci K, Colak T, Tuncar A, et al. Modified Early Warning Score and VitalPac Early Warning Score in geriatric patients admitted to emergency department. Eur J Emerg Med 2015.
(33) Ridley S. The recognition and early management of critical illness. Ann R Coll Surg Engl 2005 Sep;87(5):315-322.
(34) Aimonino Ricauda N, Tibaldi V, Leff B, Scarafiotti C, Marinello R, Zanocchi M, et al. Substitutive “hospital at home” versus inpatient care for elderly patients with exacerbations of chronic obstructive pulmonary disease: a prospective randomized, controlled trial. J Am Geriatr Soc 2008;56(3):493-500.
(35) Tibaldi V, Isaia G, Scarafiotti C, Gariglio F, Zanocchi M, Bo M, et al. Hospital at home for elderly patients with acute decompensation of chronic heart failure: a prospective randomized controlled trial. Arch Intern Med 2009;169(17):1569-1575.
(36) Worth A, Tierney AJ. Conducting research interviews with elderly people by telephone. J Adv Nurs 1993;18(7):1077-1084.
(37) Subbe CP, Kruger M, Rutherford P, Gemmel L. Validation of a modified Early Warning Score in medical admissions. QJM 2001 Oct;94(10):521-526.
(38) American College of Emergency Physicians, American Geriatrics Society, Emergency Nurses Association, Society for Academic Emergency Medicine, Geriatric Emergency Department Guidelines Task Force. Geriatric emergency department guidelines. Ann Emerg Med 2014 May;63(5):e7-25.
(39) Charlson ME, Pompei P, Ales KL, MacKenzie CR. A new method of classifying prognostic comorbidity in longitudinal studies: development and validation. J Chronic Dis 1987;40(5):373-383.
(40) Oud F, de Rooij S, Schuurman T, Duijvelaar K, van Munster B. [Predictive value of the VMS theme'Frail elderly': delirium, falling and mortality in elderly hospital patients]. Ned Tijdschr Geneeskd 2014;159:A8491-A8491.
(41) Katz S, Ford AB, Moskowitz RW, Jackson BA, Jaffe MW. Studies of illness in the aged: the index of ADL: a standardized measure of biological and psychosocial function. JAMA 1963;185(12):914-919.
(42) Herdman M, Gudex C, Lloyd A, Janssen M, Kind P, Parkin D, et al. Development and preliminary testing of the new five-level version of EQ-5D (EQ-5D-5L). Quality of Life Research 2011;20(10):1727-1736.
(43) Makai P, Brouwer WBF, Koopmanschap MA, Nieboer AP. Capabilities and quality of life in Dutch psycho-geriatric nursing homes: an exploratory study using a proxy version of the ICECAP-O. Quality of Life Research 2012;21(5):801-812.
(44) van Exel NJ, Scholte op Reimer WJ, Brouwer WB, van den Berg B, Koopmanschap MA, van den Bos GA. Instruments for assessing the burden of informal caregiving for stroke patients in clinical practice: a comparison of CSI, CRA, SCQ and self-rated burden. Clin Rehabil 2004 Mar;18(2):203-214.
(45) Robinson BC. Validation of a Caregiver Strain Index. Journal of Gerontology 1983 May 01;38(3):344-348.
(46) Bouwmans C, Hakkaart-van Roijen L, Koopmanschap M, Krol M, Severens H, Brouwer W. Handleiding iMTA Medical Cost Questionnaire (iMCQ). Rotterdam: iMTA, Erasmus Universiteit Rotterdam, 2013.
(47) Gavinski K, Carnahan R, Weckmann M. Validation of the delirium observation screening scale in a hospitalized older population. Journal of Hospital Medicine 2016:n/a-n/a.
(48) Stratton RJ, Hackston A, Longmore D, Dixon R, Price S, Stroud M, et al. Malnutrition in hospital outpatients and inpatients: prevalence, concurrent validity and ease of use of the ‘malnutrition universal screening tool’(‘MUST’) for adults. Br J Nutr 2004;92(05):799-808.

For peer review only - http://bmjopen.bmj.com/site/about/guidelines.xhtml

(49) Hawker GA, Mian S, Kendzerska T, French M. Measures of adult pain: Visual Analog Scale for Pain (VAS Pain), Numeric Rating Scale for Pain (NRS Pain), McGill Pain Questionnaire (MPQ), Short-Form McGill Pain Questionnaire (SF-MPQ), Chronic Pain Grade Scale (CPGS), Short Form-36 Bodily Pain Scale (SF-36 BPS), and Measure of Intermittent and Constant Osteoarthritis Pain (ICOAP). Arthritis Care & Research 2011;63(S11):S240-S252.
(50) de Haes,J.C., van Knippenberg,F.C., Neijt JP. Measuring psychological and physical distress in cancer patients: structure and application of the Rotterdam Symptom Checklist. Br J Cancer 1990 12;62(6):1034-1038.
(51) MacKnight C, Rockwood K. A Hierarchical Assessment of Balance and Mobility. Age Ageing 1995 Mar;24(2):126-130.
(52) Attkisson CC, Greenfield TK. The UCSF Client Satisfaction Scales: I. The Client Satisfaction Questionnaire-8. 1999.
(53) Reichheld FF. The one number you need to grow. Harv Bus Rev 2003;81(12):46-55.
(54) Quinn TJ, Dawson J, Lees JS, Chang T, Walters MR, Lees KR, et al. Time Spent at Home Poststroke: “Home-Time” a Meaningful and Robust Outcome Measure for Stroke Trials. Stroke 2008 January 01;39(1):231-233.
(55) Drummond MF, Sculpher MJ, Claxton K, Stoddart GL, Torrance GW. Methods for the economic evaluation of health care programmes. : Oxford university press; 2015.
(56) Hakkaart-van Roijen L, Van der Linden N, Bouwmans C, Kanters T, Tan S. Kostenhandleiding. Methodologie van kostenonderzoek en referentieprijzen voor economische evaluaties in de gezondheidszorg. In opdracht van Zorginstituut Nederland. Geactualiseerde versie 2015.
(57) Ritchie J, Spencer L. Qualitative data analysis for applied policy research. The qualitative researcher’s companion 2002;573(2002):305-329.
(58) Wet bescherming persoonsgegevens (2000, 6th of July). Available at: http://wetten.overheid.nl/BWBR0011468/2017-07-01/0/. Accessed October 25, 2017.
(59) Schulz KF, Altman DG, Moher D. CONSORT 2010 Statement: updated guidelines for reporting parallel group randomised trials. Trials 2010;11(1):32.
(60) Raad voor de leefomgeving en infrastructuur. Langer zelfstandig, een gedeelde opgave van wonen, zorg en welzijn. Advies van de Raad voor de Leefomgeving en infrastructuur over langer zelfstandig wonen, 2014. Available at http://www.rli.nl/sites/default/files/rli-briefadvieslangerzelfstandigeengedeeldeopgavevanwonenzorgenwelzijndef23-113-30_0.pdf Accessed October 25, 2017.
(61) Caplan GA, Sulaiman NS, Mangin DA, Aimonino Ricauda N, Wilson AD, Barclay L. A meta-analysis of "hospital in the home". Med J Aust 2012 Nov 5;197(9):512-519.
(62) Porter ME. What Is Value in Health Care? N Engl J Med 2010 12/23; 2016/06;363(26):2477-2481.
(63) Bradshaw LE, Goldberg SE, Lewis SA, Whittamore K, Gladman J, Jones R, et al. Six-month outcomes following an emergency hospital admission for older adults with co-morbid mental health problems indicate complexity of care needs. Age Ageing 2013;42(5):582-588.
(64) Sayer C. “Time Spent at Home” — A Patient-Defined Outcome. NEJM Catalyst. 2016.
(65) Groff AC, Colla CH, Lee TH. Days Spent at Home — A Patient-Centered Goal and Outcome. N Engl J Med 2016 10/27; 2017/01;375(17):1610-1612.

Figure Legends

Figure 1: Flowchart of trial design summary

For peer review only - http://bmjopen.bmj.com/site/about/guidelines.xhtml

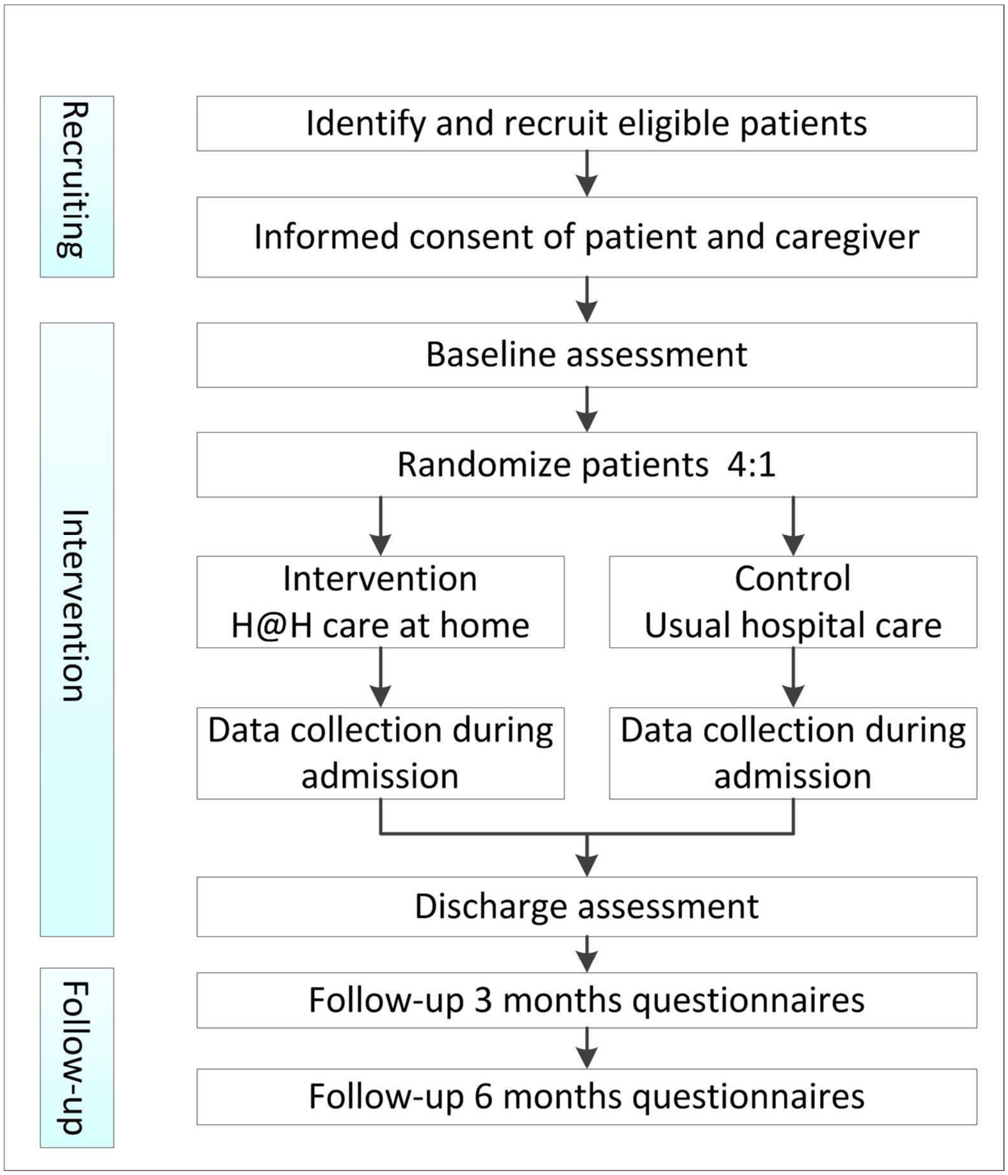

Figure 1: Flowchart of trial design summary

127x148mm (300 x 300 DPI)

STANDARD PROTOCOL ITEMS: RECOMMENDATIONS FOR INTERVENTIONAL TRIALS

SPIRIT 2013 Checklist: Recommended items to address in a clinical trial protocol and related documents*

Section/item	Item No	Description	Addressed on page number
**Administrative information**			
Title	1	Descriptive title identifying the study design, population, interventions, and, if applicable, trial acronym	1
Trial registration	2a	Trial identifier and registry name. If not yet registered, name of intended registry	1
	2b	All items from the World Health Organization Trial Registration Data Set	1, 3, 4, 5, 6, 8, 9, 13, 14
Protocol version	3	Date and version identifier	all
Funding	4	Sources and types of financial, material, and other support	14
Roles and responsibilities	5a	Names, affiliations, and roles of protocol contributors	14
	5b	Name and contact information for the trial sponsor	14
	5c	Role of study sponsor and funders, if any, in study design; collection, management, analysis, and interpretation of data; writing of the report; and the decision to submit the report for publication, including whether they will have ultimate authority over any of these activities	14
	5d	Composition, roles, and responsibilities of the coordinating centre, steering committee, endpoint adjudication committee, data management team, and other individuals or groups overseeing the trial, if applicable (see Item 21a for data monitoring committee)	n/a

For peer review only - http://bmjopen.bmj.com/site/about/guidelines.xhtml

Introduction

Background and rationale	6a	Description of research question and justification for undertaking the trial, including summary of relevant studies (published and unpublished) examining benefits and harms for each intervention	3
	6b	Explanation for choice of comparators	3
Objectives	7	Specific objectives or hypotheses	3
Trial design	8	Description of trial design including type of trial (eg, parallel group, crossover, factorial, single group), allocation ratio, and framework (eg, superiority, equivalence, noninferiority, exploratory)	4

Methods: Participants, interventions, and outcomes

Study setting	9	Description of study settings (eg, community clinic, academic hospital) and list of countries where data will be collected. Reference to where list of study sites can be obtained	4
Eligibility criteria	10	Inclusion and exclusion criteria for participants. If applicable, eligibility criteria for study centres and individuals who will perform the interventions (eg, surgeons, psychotherapists)	6
Interventions	11a	Interventions for each group with sufficient detail to allow replication, including how and when they will be administered	7, 8
	11b	Criteria for discontinuing or modifying allocated interventions for a given trial participant (eg, drug dose change in response to harms, participant request, or improving/worsening disease)	7, 8
	11c	Strategies to improve adherence to intervention protocols, and any procedures for monitoring adherence (eg, drug tablet return, laboratory tests)	n/a
	11d	Relevant concomitant care and interventions that are permitted or prohibited during the trial	9, 10
Outcomes	12	Primary, secondary, and other outcomes, including the specific measurement variable (eg, systolic blood pressure), analysis metric (eg, change from baseline, final value, time to event), method of aggregation (eg, median, proportion), and time point for each outcome. Explanation of the clinical relevance of chosen efficacy and harm outcomes is strongly recommended	9, 10
Participant timeline	13	Time schedule of enrolment, interventions (including any run-ins and washouts), assessments, and visits for participants. A schematic diagram is highly recommended (see Figure)	10

For peer review only - http://bmjopen.bmj.com/site/about/guidelines.xhtml

Sample size	14	Estimated number of participants needed to achieve study objectives and how it was determined, including clinical and statistical assumptions supporting any sample size calculations	6
Recruitment	15	Strategies for achieving adequate participant enrolment to reach target sample size	5
**Methods: Assignment of interventions (for controlled trials)**			
Allocation:			
Sequence generation	16a	Method of generating the allocation sequence (eg, computer-generated random numbers), and list of any factors for stratification. To reduce predictability of a random sequence, details of any planned restriction (eg, blocking) should be provided in a separate document that is unavailable to those who enrol participants or assign interventions	4
Allocation concealment mechanism	16b	Mechanism of implementing the allocation sequence (eg, central telephone; sequentially numbered, opaque, sealed envelopes), describing any steps to conceal the sequence until interventions are assigned	4
Implementation	16c	Who will generate the allocation sequence, who will enrol participants, and who will assign participants to interventions	4
Blinding (masking)	17a	Who will be blinded after assignment to interventions (eg, trial participants, care providers, outcome assessors, data analysts), and how	4
	17b	If blinded, circumstances under which unblinding is permissible, and procedure for revealing a participant's allocated intervention during the trial	n/a
**Methods: Data collection, management, and analysis**			
Data collection methods	18a	Plans for assessment and collection of outcome, baseline, and other trial data, including any related processes to promote data quality (eg, duplicate measurements, training of assessors) and a description of study instruments (eg, questionnaires, laboratory tests) along with their reliability and validity, if known. Reference to where data collection forms can be found, if not in the protocol	9, 10, 11
	18b	Plans to promote participant retention and complete follow-up, including list of any outcome data to be collected for participants who discontinue or deviate from intervention protocols	11

For peer review only - http://bmjopen.bmj.com/site/about/guidelines.xhtml

Data management	19	Plans for data entry, coding, security, and storage, including any related processes to promote data quality (eg, double data entry; range checks for data values). Reference to where details of data management procedures can be found, if not in the protocol	11
Statistical methods	20a	Statistical methods for analysing primary and secondary outcomes. Reference to where other details of the statistical analysis plan can be found, if not in the protocol	12
	20b	Methods for any additional analyses (eg, subgroup and adjusted analyses)	n/a
	20c	Definition of analysis population relating to protocol non-adherence (eg, as randomised analysis), and any statistical methods to handle missing data (eg, multiple imputation)	n/a
**Methods: Monitoring**			
Data monitoring	21a	Composition of data monitoring committee (DMC); summary of its role and reporting structure; statement of whether it is independent from the sponsor and competing interests; and reference to where further details about its charter can be found, if not in the protocol. Alternatively, an explanation of why a DMC is not needed	12
	21b	Description of any interim analyses and stopping guidelines, including who will have access to these interim results and make the final decision to terminate the trial	12
Harms	22	Plans for collecting, assessing, reporting, and managing solicited and spontaneously reported adverse events and other unintended effects of trial interventions or trial conduct	12
Auditing	23	Frequency and procedures for auditing trial conduct, if any, and whether the process will be independent from investigators and the sponsor	n/a
**Ethics and dissemination**			
Research ethics approval	24	Plans for seeking research ethics committee/institutional review board (REC/IRB) approval	12
Protocol amendments	25	Plans for communicating important protocol modifications (eg, changes to eligibility criteria, outcomes, analyses) to relevant parties (eg, investigators, REC/IRBs, trial participants, trial registries, journals, regulators)	12

For peer review only - http://bmjopen.bmj.com/site/about/guidelines.xhtml

Consent or assent	26a	Who will obtain informed consent or assent from potential trial participants or authorised surrogates, and how (see Item 32)	5
	26b	Additional consent provisions for collection and use of participant data and biological specimens in ancillary studies, if applicable	n/a
Confidentiality	27	How personal information about potential and enrolled participants will be collected, shared, and maintained in order to protect confidentiality before, during, and after the trial	10
Declaration of interests	28	Financial and other competing interests for principal investigators for the overall trial and each study site	13
Access to data	29	Statement of who will have access to the final trial dataset, and disclosure of contractual agreements that limit such access for investigators	10
Ancillary and post-trial care	30	Provisions, if any, for ancillary and post-trial care, and for compensation to those who suffer harm from trial participation	Available on request
Dissemination policy	31a	Plans for investigators and sponsor to communicate trial results to participants, healthcare professionals, the public, and other relevant groups (eg, via publication, reporting in results databases, or other data sharing arrangements), including any publication restrictions	12
	31b	Authorship eligibility guidelines and any intended use of professional writers	13
	31c	Plans, if any, for granting public access to the full protocol, participant-level dataset, and statistical code	n/a
**Appendices**			
Informed consent materials	32	Model consent form and other related documentation given to participants and authorised surrogates	Available on request
Biological specimens	33	Plans for collection, laboratory evaluation, and storage of biological specimens for genetic or molecular analysis in the current trial and for future use in ancillary studies, if applicable	n/a

*It is strongly recommended that this checklist be read in conjunction with the SPIRIT 2013 Explanation & Elaboration for important clarification on the items. Amendments to the protocol should be tracked and dated. The SPIRIT checklist is copyrighted by the SPIRIT Group under the Creative Commons "Attribution-NonCommercial-NoDerivs 3.0 Unported" license.

For peer review only - http://bmjopen.bmj.com/site/about/guidelines.xhtml

BMJ Open

Hospital at Home care for older patients with cognitive impairment: A protocol for a randomized controlled feasibility trial

Journal:	*BMJ Open*
Manuscript ID	bmjopen-2017-020332.R2
Article Type:	Protocol
Date Submitted by the Author:	08-Feb-2018
Complete List of Authors:	Pouw, Maaike; University of Groningen, University Medical Center Groningen, Department of Geriatrics; Martini Hospital, Department of Internal Medicine<br>Calf, Agneta; University of Groningen, University Medical Center Groningen, Department of Geriatrics<br>van Munster, Barbara; University of Groningen, University Medical Center Groningen, Department of Geriatrics; Gelre Hospitals, Department of Geriatrics<br>ter Maaten, Jan; University of Groningen, University Medical Center Groningen, Department of Internal Medicine, Emergency Department<br>Smidt, Nynke; University of Groningen, University Medical Center Groningen, Department of Epidemiology, The Netherlands<br>De Roolj, Sophia; University of Groningen, University Medical Center Groningen, Department of Geriatrics
**Primary Subject Heading**:	Geriatric medicine
Secondary Subject Heading:	Patient-centred medicine
Keywords:	Dementia < NEUROLOGY, Organisation of health services < HEALTH SERVICES ADMINISTRATION & MANAGEMENT, GERIATRIC MEDICINE, Delirium & cognitive disorders < PSYCHIATRY, INTERNAL MEDICINE

SCHOLARONE™
Manuscripts

Public title

Hospital at Home care for older patients with cognitive impairment and an acute medical illness

Scientific title

Hospital at Home care for older patients with cognitive impairment: A protocol for a randomized controlled feasibility trial

Acronym

H@H

Maaike A Pouw,[1,2] Agneta H Calf,[1] Barbara C van Munster,[1,3] Jan C ter Maaten,[4] Nynke Smidt,[1,5] & Sophia E de Rooij [1]

Corresponding author: m.a.pouw@umcg.nl. University Center of Geriatric Medicine, University Medical Center Groningen. Hanzeplein 1, Postbus 30 001, 9700 RB Groningen

Authors' affiliations

[1] Department of Geriatrics, University Medical Center Groningen, University of Groningen, Groningen, the Netherlands

[2] Department of Internal Medicine, Martini Hospital, Groningen, The Netherlands.

[3] Department of Geriatrics, Gelre Hospitals, Apeldoorn, The Netherlands.

[4] Department of Internal Medicine, Emergency Department, University Medical Center Groningen, University of Groningen, Groningen, The Netherlands

[5] Department of Epidemiology, University of Groningen, University Medical Center Groningen, Groningen, The Netherlands.

Keywords: Dementia, Cognition disorders, Aged, Home Care Services; Hospital-Based, Hospital at Home

Word count: 3985

For peer review only - http://bmjopen.bmj.com/site/about/guidelines.xhtml

Abstract

INTRODUCTION: An acute hospital admission is a stressful life event for older people, particularly for those with cognitive impairment. The hospitalisation is often complicated by hospital-associated geriatric syndromes, including delirium and functional loss, leading to functional decline and nursing home admission. Hospital at Home care aims to avoid hospitalisation-associated adverse outcomes in older patients with cognitive impairment by providing hospital care in the patient's own environment.

METHODS AND ANALYSIS: This randomized, non-blinded feasibility trial aims to assess the feasibility of conducting a randomized controlled trial in terms of the recruitment, use and acceptability of Hospital at Home care for older patients with cognitive impairment. The quality of care will be evaluated and the advantages and disadvantages of the Hospital at Home care program compared to usual hospital care. Eligible patients will be randomized either to Hospital at Home care in their own environment or usual hospital care. The intervention consists of hospital level care provided at patients' homes, including visits from health care professionals, diagnostics (laboratory tests, blood cultures) and treatment. The control group will receive usual hospital care. Measurements will be conducted at baseline, during admission, at discharge and at three and six months after the baseline assessment.

ETHICS AND DISSEMINATION: Institutional ethics approval has been granted. The findings will be disseminated through public lectures, professional and scientific conferences, as well as peer-reviewed journal articles. The study findings will contribute to knowledge on the implementation of Hospital at Home care for older patients with cognitive disorders. The results will be used to inform and support strategies to deliver eligible care to older patients with cognitive impairment.

TRIAL REGISTRATION: This study was registered with the NTR registry (NTR6581) on 26 July 2017.

For peer review only - http://bmjopen.bmj.com/site/about/guidelines.xhtml

Strengths and limitations of this study

- This study addresses the feasibility of Hospital at Home care in patients with cognitive impairment, a patient population that is often excluded from participation in scientific research
- A process evaluation facilitates the investigation of factors that influence the experiences and perceptions of all persons involved in Hospital at Home care
- Stakeholders were involved in the development of the design of the study which will support the implementation of Hospital at Home care and a future trial
- Because of a limited sample size due to the study being centered on feasibility, results will not show effectiveness of Hospital at Home care compared to usual hospital care.

Introduction

Background

An acute hospital admission is a stressful life event, particularly for older people. In addition to the stress of an acute illness, the hospital admission itself contributes to this stress.[1] Older hospitalized patients are often deprived of sleep, and they spend an average of 20 of every 24 hours in bed, they become poorly nourished, and experience sensory deprivation or overstimulation, resulting in confusion.[2-5] These adverse effects of hospitalization contribute to the occurrence of geriatric conditions, such as delirium, functional decline, falls, incontinence, hospital acquired infections and pressure injuries.[6-9] Adverse effects of hospitalization occur more easily in older people, particularly in those who are already frail, a growing portion of the worldwide ageing population.[10,11] Frailty is a state of increased vulnerability to external stressors resulting from aging-associated declines in reserve and function across multiple physiologic systems.[10] Cognitive impairment (i.e., dementia) is an important contributor to frailty in older people.[12] Cognitively impaired older people are more likely to become hospitalised and once admitted, they experience longer stays than their peers without cognitive impairment.[13-15] The combination of hospitalization and cognitive impairment in older people is associated with further functional and cognitive declines and higher mortality rates, and it leads to more discharges to long-term care facilities.[16,17] The prevalence and worldwide burden of cognitive impairment will continue to increase as the average life expectancy increases.[18] The total number of people with cognitive impairment is estimated to be 75.6 million in 2030 and will nearly triple in 2050 to 135.5 million.[19] An increase in the number of hospital admissions of older people with cognitive impairment and an increase in number of hospitalisation-associated adverse outcomes are therefore to be expected.

For peer review only - http://bmjopen.bmj.com/site/about/guidelines.xhtml

Besides adverse outcomes of hospitalization, many older people and their caregivers do not necessarily desire a hospital admission in case of an acute illness or exacerbation of a chronic illness. Fried et al. (2000) have studied the preferences of community-dwelling persons 65 years of age and older who were hospitalized with a primary diagnosis of congestive heart failure, chronic obstructive pulmonary disease or pneumonia. The authors reported that over 50% of older patients preferred to receive hospital treatment at home, because they felt that their homes were more comfortable.[20] In the treatment preferences of seriously ill patients 60 years of age and older, the likelihood of cognitive and functional impairment as an adverse outcome of the treatment was weighed in the decision-making process. There was a substantial decrease in the number of participants who opted for treatment if the likelihood of impairment after treatment was 50% or higher.[21]

Hospital at Home care could provide an effective alternative to inpatient care for a select group of elderly patients now requiring hospitalisation. Hospital at Home care is coordinated, multidisciplinary care in the homes of people who would otherwise be admitted to the hospital. Hospital at Home care is an accepted alternative to inpatient hospital level care in several countries (e.g., the United States, Australia, Italy and the United Kingdom) but not yet in the Netherlands.[22] Since the 1990s, Hospital at Home has been evaluated in (older) persons with various acute medical conditions, such as heart failure, exacerbations in chronic obstructive pulmonary disease (COPD) and infections (e.g., cellulitis, pneumonia).[22] In systematic reviews comparing alternative strategies to inpatient hospitalization, lower or equal mortality rates and return hospitalization rates (i.e., subsequent admissions after discharge) were found for Hospital at Home care, there was a lower incidence of delirium, and there was a positive effect on patient and caregiver satisfaction.[22-24] Only one completed trial conducted in Italy included 109 patients with cognitive impairment (i.e. dementia), Tibaldi et al. reported a positive effect of a Hospital at Home intervention on behavioural disturbances and caregiver stress in patients with dementia.[25] Results of a still on-going trial including people with i.a. cognitive impairment in the United Kingdom, will follow in the near future.[26] Whether Hospital at Home care provides a suitable alternative with regard to other outcomes as patient satisfaction, quality of care, hospitalisation-associated adverse events and costs in older people with cognitive impairment remains unclear and further research is needed. Therefore, our primary aim is to investigate the feasibility of a Hospital at Home care program for older patients with cognitive impairment in terms of the patient recruitment, use and acceptability, and secondly to investigate the advantages and disadvantages of Hospital at Home care compared to usual hospital care from different perspectives.

Objectives of this study are:

For peer review only - http://bmjopen.bmj.com/site/about/guidelines.xhtml

1) To assess the participation rate of the Hospital at Home trial among patients 65 years and older with cognitive impairment, acute illness, and emergency hospital admission. What are the reasons for non-participation?

2) To assess the potential advantages and disadvantages of Hospital at Home care and usual hospital care for the patients, caregivers and Dutch medical health system.

3) To assess the feasibility of Hospital at Home care in terms of the quality of care with regard to geriatric syndromes, institutionalization, mortality, total days with urinary catheter, length of stay (in the hospital or in Hospital at Home care) and timing/intensity of the contact with health care professionals.

Methods and analysis

Trial design

The design is a randomized controlled feasibility trial and will utilize a process evaluation. This study will be conducted at the medical emergency department (ED) of the academic hospital of the University Medical Center of Groningen in the Netherlands and will evaluate cognitively impaired older patients who are in need of acute hospital care. Figure 1 shows the trial design summary. Participants will be randomized to either Hospital at Home care or usual hospital care in a 4:1 ratio, respectively. Patients will be randomized using a computerized random number generator (http://www.randomization.com), including block randomization.

An independent research nurse who is not involved in the patient care will complete the baseline assessment and allocate the participants (using sealed sequenced envelopes) into the Hospital at Home care (intervention) or usual hospital care group (control). The research nurse will not be aware of the randomization method. The participants, health care professionals and research staff will not be blinded to the intervention. The reporting of the design of this trial protocol is in accordance with the SPIRIT 2013 statement for clinical trial protocols.[27]

For peer review only - http://bmjopen.bmj.com/site/about/guidelines.xhtml

Figure 1

Study population

Patients 65 years of age and older who are admitted to the medical emergency department (ED) will be identified by the ED staff as potential eligible patients. Subsequently, the ED staff will inform the research nurse. The research nurse will complete the eligibility criteria checklist (table 1). The research nurse will ask the patient and their informal caregiver (i.e., partner, child, relative, friend) for their willingness to participate in the study and to provide written informed consent. The patient and informal caregiver will need to both sign the informed consent form before the patient can participate in the H@H trial.

An evaluation to assess the mental capacity is conducted by the involved ED staff in the setting of the emergency department assessment. If the participant lacks the capacity to consent (mentally incapacitated), and an informal caregiver (i.e., partner, child, friend) is present, this informal caregiver will be asked to act as a personal consultee. The personal consultee will determine whether he or she believes that participation in the study would be in accordance with the values and interests of the individual and will subsequently sign the patient's informed consent form.

view only

For peer review only - http://bmjopen.bmj.com/site/about/guidelines.xhtml

Table 1 Patient eligibility criteria for participation in Hospital at Home trial

Inclusion criteria	Exclusion criteria
▪ Age 65 years of age and older<br>▪ Cognitive impairment, i.e. dementia, delirium or other cause of cognitive impairment, and either:<br>○ previously diagnosed or documented in the medical records or<br>○ identified by the ED-clinician (e.g., with the 4AT-test and/or Six item cognitive impairment test)[28-30]<br>▪ Presented at the ED with a defined acute illness<br>▪ Required hospital admission, according to the attending ED physician but not expected to require emergency interventions<br>○ Modified Early Warning Score (MEWS) ≤ 2 points[31-33]<br>▪ Living in hospital's catchment area (< 25 km)<br>▪ Informal caregiver is present and able to understand and perform instructions and consented to participate in the trial<br>▪ Home suitable for Hospital at Home care (available informal caregiver, running water, adequate heating, safety) [30]	▪ Previously enrolled<br>▪ Hospitalized within the seven days preceding ED presentation<br>▪ Nursing home residents or awaiting a nursing home place on an active waiting list (excluding so-called sleeping waiting list candidates)[27]<br>▪ Additional care needed<br>○ Required surgical assessment<br>○ Suspected acute coronary syndrome or cardiac arrythmia[31]<br>○ Dialysis dependent patients[31]<br>○ Expected terminal events[31] or in need of diagnostic or palliative care due to oncological or haematological illness

ED= Emergency department

Sample size of study population

Based on the numbers available from the emergency department (ED) of the University Medical Center Groningen, the Netherlands, we calculated that an average of 3990 older patients 65 years and over is admitted to our medical ED each year. The Hospital at Home trial will be introduced during working hours, which provides an estimated 1900 patients per year. Not all 1900 patients will be eligible for study inclusion. Based on screening of ED medical records, approximately 15% of the patients meet the eligibility criteria for Hospital at Home care, resulting in 285 eligible persons per year. In recent randomized controlled trials (RCTs) of Hospital at Home care in Italy, 54% and 57% of the eligible patients was willing to participate and gave informed consent.[34,35] We presume a similar consent rate of 50%, as described in these previous clinical trials, and expect for 143 patients to be included.

Study procedures

After (written) informed consent is obtained, all participants will complete two brief tests to assess cognitive impairment, and the participant and caregiver will complete the baseline assessment. Subsequently, randomization takes place to either (a) the Hospital at Home care-intervention, translocation of care from the hospital to a participants' home or (b) the control group, usual hospital care. All care will be delivered according to hospital protocols, current regulations and

For peer review only - http://bmjopen.bmj.com/site/about/guidelines.xhtml

guidelines and, if needed, described in the standard operating procedures (SOPs). If no informed consent is given by either the patient or the caregiver, the reasons for non-participation, date of birth, sex of the patient and the relationship between patient and caregiver will be reported.

Intervention

Hospital at Home care

Hospital at Home care will be delivered by a multidisciplinary team consisting of a physician, nurse, pharmacist and physiotherapist. Depending on the participants' needs, other disciplines (e.g., a dietician, occupational therapist or social services) can be involved in the Hospital at Home care. The day-to-day care will be provided by the nurse and physician visiting the participant. The Hospital at Home care team works under the responsibility of the medical specialist in the hospital, and 24/7 consultation of the expertise and services of the hospital is part of the protocol.

The participants allocated to Hospital at Home care will receive hospital level care in their own homes. After a stay of one night in the hospital, while the Hospital at Home care arrangements are being made, the participant will be transferred home and receive Hospital at Home care. The Hospital at Home nurse is responsible for the day-to-day care and will be present upon arrival of the participant at his/her residence. Hospital at Home care is described in the care protocols including SOPs and could include intravenous therapy (e.g., antibiotics, fluid, and/or diuretics), oxygen therapy, and/or nebulizer, indwelling urine catheter or a nasopharyngeal food tube. After the care intake and a period of direct nursing supervision, the participant will receive intermittent nursing visits daily (starting with 3x/day), including weekends and public holidays. The Hospital at Home physician will make a home visit every day (excl. weekends). The Hospital at Home physician and nurse will be available for emergency visits. The participant will receive a medical alert device in the house, with a 24/7 connection to an on-call service. Alert instructions will be explained to the participant and caregiver. A physiotherapist will visit the participant at home to evaluate any problems with balance and/or walking and immobility. The Hospital at Home team works under the supervision of the hospital medical specialist. Daily screenings and measurements will be recorded in a Hospital at Home record, which stays with the participant. Diagnostic procedures and therapeutics that cannot wait and are not available at home, such as endoscopy or CT-scan, will be arranged through brief visits to the hospital. The participant will be 'admitted' to the Hospital at Home care for as long as indicated.

Discharge from Hospital at Home

If the participant recovers to such an extent that hospital level care is no longer needed, the participant will be discharged from the Hospital at Home care program, similar to the discharge

For peer review only - http://bmjopen.bmj.com/site/about/guidelines.xhtml

procedure when the participant would receive usual hospital care. Hospital at Home care will end with discharge planning with the participant, family, Hospital at Home physician and nurse. The discharge plan includes follow-up appointments (e.g., at the hospital or general practitioner), information on medication, warning signs and symptoms and an on-going management plan. All hospital-related care equipment will be removed from the participants' house, and arrangements with homecare agencies and/or paramedical staff will be reviewed and adjusted to the current situation.

Hospital care as usual

Participants allocated to the control arm will be admitted to a hospital ward and receive usual hospital care. After admission and intake on the ward, the participant will receive intermittent visits from the ward nurse multiple times a day. The ward physician will visit once every day (excl. weekends), with extra visits provided if needed. An emergency alert device, through which nurses and physician can be contacted, will be placed next to the bed. A physiotherapist will visit the participant at the ward to address problems with balance and/or walking and immobility. Depending on the participants' needs, other disciplines (e.g., a dietician, occupational therapist or social services) can be involved in the hospital care. The medical record is the hospital record and additional H@H research forms will be added to this record for research purposes. The participant will be admitted to the hospital for as long as indicated.

Hospital discharge

If the participant recovers to such an extent that hospital level care is no longer needed, the participant will be discharged from the hospital after discharge planning with the participant, family, physician and nurse. The discharge plan includes follow-up appointments (e.g., at the hospital or general practitioner), information on medication, warning signs and symptoms and an on-going management plan. Arrangements with homecare agencies and/or paramedical staff will be reviewed and adjusted to the current situation.

Follow-up

At three and six months following randomization, all participants will be contacted for an interview by telephone[36] or a face-to-face interview, if needed. The participants will be allowed to receive support with these questions from their relatives or informal caregivers. An interview will require a maximum of 30 minutes. In case of institutionalization or mortality, this event will be recorded. Additionally, information on hospital readmission and length of stay will be collected from the

For peer review only - http://bmjopen.bmj.com/site/about/guidelines.xhtml

hospital administration system and health insurers. Mortality and nursing home placements will be collected from registries from the general practitioner and municipalities.

Timing of measurements and outcome measures

Data will be collected at baseline at the emergency department, during admission (in Hospital at Home or hospital), at discharge and at three and six months following randomization, plus or minus two weeks. An overview of the timing of measurements and outcome measures are shown in Table 2.

Feasibility

For the participation rates, the proportion of participants per step will be calculated. The reasons for non-participation and data concerning the characteristics of non-participants will be collected. We consider the participation rate feasible when it is similar to the participation rate as is described in previous RCTs and around 50% of the eligible patients will consent to participate.[34,35] Quality of care will be measured by collecting data on patient, institutionalization (e.g., to the hospital or nursing home), mortality, ADL-functioning, prevalence of hospitalisation-associated geriatric syndromes, the length of stay in the hospital or Hospital at Home care program and contact with health care professionals. The study is considered feasible if the quality of care of Hospital at Home care on these measurements is non- inferior to usual hospital care.

For peer review only - http://bmjopen.bmj.com/site/about/guidelines.xhtml

Table 2 Overview of the content and description of outcome measures and timing of measurements

	Description and instrument	Timing of measurements					
		screening	baseline	admission	discharge	3 months	6 months
Degree of illness based on physiological parameters	Vital signs alarm score; Modified Early Warning Score, [37]	R					
Cognitive impairment	4AT-test for delirium* Six item cognitive impairment test for cognitive impairment*,[28,38]	R					
Socio-demographics	Date of birth, nationality, household composition, marital status, highest level of education		R				
Health status	Charlson co-morbidity index*,[39]		R				
Identifying at-risk patients	Safety management system patient screening (VMS),[40]		R				
Functional status	Activities of daily living, modified Katz-ADL index score,[41]		R		R	R	R
Health status	EQ-5d-5l*,[42]		R		R	R	R
(Health-related) quality of life, well-being	Icepop capability measure for Older people (ICECAP-O)*[43]		R		R	R	R
Caregiver burden	Self-rated burden scale*, caregiver strain index*,[44,45]		C		C	C	C
Medical consumption	Imta Medical Consumption Questionnaire (imcq)*,[46]		P,C			P,C	P,C
Hospitalisation-associated geriatric syndromes	Infections, falls, pressure injuries, in case of delirium; delirium observation scale score (DOSS)[47] and use of physical or chemical restraints, total days with a urinary catheter			N			
Nutrition	Malnutrition Universal Screening Tool (MUST),[48]		N				
	Food intake, fluid intake			N			
Pain	Numeric Rating Scale-score (NRS) for pain [49]			N			
Health perception	(Rotterdam) symptom checklist*[50]			N			
Immobility	Hierarchical assessment of balance and mobility (HABAM)[51]			Ph			
Satisfaction with care	Client Satisfaction Questionnaire 8 (CSQ-8)*, care evaluation question*,[52,53]				P<br>P,C,<br>N,D		
Mortality	Mortality at 30 days, 3 months and 6 months after baseline*				N	R	R
(Re)admission hospital	Length of stay, readmission rate at 30 days, 3 months and 6 months after baseline*				N	R	R

Assessed by: P= Participant, C=Caregiver, N= Nurse, D= Doctor, R= Research nurse, Ph=Physiotherapist.
(*)= All assessments marked by an asterisk are extra for trial purposes and are not part of the medical treatment

Other outcomes measures

Advantages and disadvantages of the Hospital at Home care program will be assessed through multiple instruments and questionnaires. The instruments are validated and used in community-dwelling older patients with cognitive disorders. Additional data will be collected on the time spent at home (home-time); total number of days alive and out of the hospital or a skilled nursing facility in the 6 months following the randomization at the emergency department,[54] the number of transfers (home ↔ hospital) and the number of health care professionals involved.

For peer review only

For peer review only - http://bmjopen.bmj.com/site/about/guidelines.xhtml

Cost data will be collected, as described by Drummond et al., including the costs to the health care system, patients and families, and other sectors.[55] The volume of care use will be extracted from hospital files and combined with the reference cost values, as provided by the cost guidance module of the Dutch National Health Care Institute.[56]

Process evaluation

A process evaluation will be conducted as part of the feasibility study to understand the barriers and facilitators to participate and to gain an understanding of the experiences and perceptions of Hospital at Home care of participants and health care professionals. From all eligible patients who declined to participate, data concerning the patient characteristics and reasons for non-participation will be collected. At the end of the trial, a representative sample of participants and/or their informal caregivers will be invited for an interview to evaluate their experiences receiving Hospital at Home care. The interviewer will not be a an active member of the research team or involved in day-to-day care and will explore independently how the participants perceived Hospital at Home care, including the contact with the health professionals and the impact of Hospital at Home care on their lives and their caregivers' personal lives. In case of participant dropout, efforts will be made to obtain an understanding of why the participants did not complete the trial.

In addition, a representative sample of health care professionals, consisting of physicians and nurses working in the Emergency Department, physicians and nurses providing the Hospital at Home care, and general practitioners, will be asked to participate in a face-to-face interview. The health care professionals will be asked about their experiences and opinions about the H@H-trial and Hospital at Home care. All interviews will be transcribed verbatim, and a framework analysis will be used as the method of qualitative data analysis.[57]

Data management

All data will be entered in an electronic trial-specific database, with the participants identified by a unique trial number. Confidentiality of participant information will be maintained throughout the trial. Information can only be traced to the participants by designated researchers. The database will be stored and maintained by Castor Electronic Data Capture, compliant with GCP guidelines and the European Data Protection Directive (Castor Electronic Data Capture, Ciwit BV, Amsterdam, the Netherlands, 2017). Data will be stored for a maximum period of 15 years after the study has ended, according to Dutch law.[58]

For peer review only - http://bmjopen.bmj.com/site/about/guidelines.xhtml

Statistical analysis

The participant flow diagram, according to CONSORT guidelines,[59] will provide a summary of the recruitment and declination rates in percentage (%) at baseline, discharge and three- and six-month follow-ups. Distributions of the data at baseline, discharge and three and six months after randomization will be explored, with unusual values noted and explained. Variables will be summarized as the n (%), mean (standard deviation) or median (interquartile range) for each group, to characterize the sample and search for any imbalances. The percentages, means and standard deviation, and medians and interquartile ranges will be calculated to describe the quality of care and the advantages and disadvantages of Hospital at Home care at baseline, discharge and three- and six-month follow-ups.

Monitoring and participant safety

Although, the H@H trial is considered to be a low risk trial, the participant safety will be monitored by an independent Data Monitoring Committee (DMC). The DMC will consist of two members: an experienced clinician and an epidemiologist. Members of the DMC are independent of the trial and will discuss each individual participant with serious adverse events. The DMC will receive and review the serious adverse events and evaluate the risk involved with negative outcomes. The DMC is authorized to make recommendations to temporary put on hold or ending the study prematurely when participant safety is an issue, based on their findings. All serious adverse events will be reported to the principal investigator within 24 hours of knowledge of the event and then subsequently reported to the Dutch portal for medical research involving human subjects.

Discussion

Reducing unwanted hospital admissions in older patients with cognitive impairments and facilitating patient-centred care in a patient's preferred location is a goal worthy of pursuing. This goal aligns with the tenet of the current Dutch government and the advice provided by the Dutch Council for the Environment and Infrastructure: to actively promote and enable people to live independently in their own homes for as long as they desire.[60] Previous trials and a recent review have confirmed that alternative management strategies for low-risk patients with acute medical conditions conventionally treated through hospitalization exist with positive impact on patient satisfaction, are effective and can be safely achieved in lower cost settings.[22,23,61]

For peer review only - http://bmjopen.bmj.com/site/about/guidelines.xhtml

Introducing a Hospital at Home care trajectory in the Netherlands is incited by the principles of value-based health care: improving the patients experience of care and as a result of this process reducing the costs.[62] All countries with an ageing population experience pressure, in terms of shortage of (emergency) hospital beds and rising healthcare costs. Hospital at Home care could be shown beneficial in facilitating higher valued care for patients and their caregivers without additional costs. Benefit should be measured in other outcomes than clinical indicators such as mortality. To illustrate, one of the outcomes of a future RCT could be the time spent at home. Time spent at home has been defined as the total number of days alive and out of the hospital or a skilled nursing facility in the 6 months after hospital admission.[54] It has been used as a primary outcome in a follow-up study of older patients with acute hospital admissions, and has been demonstrated to be of more importance in older patients.[63-65] Evaluation of time spent a home in this feasibility study could support estimating a sample size based on a patient-relevant outcome in a future RCT.

This study will be the first to investigate the feasibility of providing acute hospital care at home for older patients with cognitive impairment in the Netherlands. Studying Hospital at Home care and identifying the barriers and facilitators will support the implementation of Hospital at Home care and break new ground for a future RCT investigating the (cost-)effectiveness.

Ethics and Dissemination

This study was approved by the Medical Research Ethics Committee of the University Medical Center Groningen in April 2017, reference number: 2016.686 The trial will be conducted in accordance with the Declaration of Helsinki 1996, principles of good clinical practice and the University Medical Center of Groningen Research Code. Any protocol amendments will be submitted to the ethics committee. A register of the protocol amendments will be available in the study protocol.

The results of the trial will be reported according to the CONSORT guidelines and will contribute to knowledge of the implementation of Hospital at Home care and patient-centred acute care for older patients with cognitive impairment. The study will also contribute to the knowledge of the transmural cooperation and costs of providing care, in terms of the translocation of hospital care to home. Regularly updates will be published on the study website and in newsletters. Conferences and meetings will be held for all involved health care professionals. Participants who requested information on the study will be sent a lay summary. A publication policy will be agreed upon with co-applicants. The study findings will be published in relevant peer-reviewed journals.

Acknowledgements The authors would like to thank all care professionals of Alzheimer Nederland, Espria, Icare, TSN, Buurtzorg, umcGroningen thuis, MCZ, Netwerk Dementie Groningen, Netwerk Dementie Drenthe,

For peer review only - http://bmjopen.bmj.com/site/about/guidelines.xhtml

Gemeente Groningen, WIJ Groningen, Zorgbelang, Healthy Aging Network Northern Netherlands, Stichting Effectieve Ouderenzorg, ZonMW, Deltaplan Dementie, Nationaal Programma Ouderenzorg, Zorg innovatie Forum and the elderly of Denktank 60+ Noord for their time, critical appraisal and advise.

Contributors MP contributed to the conception and design of the protocol, drafted the manuscript, and revised the final manuscript. AC, BM, JM, and NS made substantial contributions to the conception and design of the protocol. NS contributed to the methodological aspects of the protocol. AC, BM and JM contributed to the clinical aspects of the protocol. SR conceived the study, developed the protocol together with MP and wrote the funding applications. All authors critically revised the manuscript and approved the final version of this manuscript.

Funding This study is independent research funded by ZonMw (project Memorabel, Deltaplan Dementie), project number #733050401 (contact: memorabel@zonmw.nl). Additional funding is provided by the Dutch Ministry of Health, Welfare and Sport (Ministerie van Volksgezondheid, Welzijn en Sport) and the University Medical Center Groningen.

Disclaimer The views and opinions expressed in this paper are those of the authors and not necessarily those of the UMCG, Memorabel, Deltaplan Dementie or ZonMW.

Competing interests All authors have declared that they have no competing interests

Ethics approval for this study has been obtained from the Medical Research Ethics Committee of the University Medical Center Groningen April 2017 (ref: 2016.686).

Recruitment status is pending; participants are not yet being recruited or enrolled. Anticipated date of enrolment of the first participant is December 2017.

Scientific inquiries: prof Sophia E.de Rooij, s.e.j.a.de.rooij@umcg.nl, telephone number 0031-50-3613921, Hanzeplein 1, Postbus 30 001, 9700 RB Groningen. University of Groningen, University Medical Center Groningen, University Center for Geriatric Medicine, Groningen, The Netherlands.
Generic email address hospitalathome@umcg.nl.

Abbreviations

ADL: Activities of Daily Living
CT: Computer Tomography
DMC: Data Monitoring Committee
ED: Emergency Department
H@H: Hospital at Home
RCT: Randomized Controlled Trial
UMCG: University Medical Center Groningen

References

(1) Krumholz HM. Post-Hospital Syndrome - A Condition of Generalized Risk. N Engl J Med 2013 01/10;368(2):100-102.
(2) Missildine K, Bergstrom N, Meininger J, Richards K, Foreman MD. Sleep in Hospitalized Elders: A Pilot Study. Geriatr Nurs 2010 0;31(4):263-271.
(3) Brown CJ, Redden DT, Flood KL, Allman RM. The underrecognized epidemic of low mobility during hospitalization of older adults. J Am Geriatr Soc 2009 Sep;57(9):1660-1665.
(4) Sullivan DH, Sun S, Walls RC. Protein-energy undernutrition among elderly hospitalized patients: a prospective study. JAMA 1999 Jun 2;281(21):2013-2019.
(5) Creditor MC. Hazards of hospitalization of the elderly. Ann Intern Med 1993;118(3):219-223.
(6) Inouye SK, Studenski S, Tinetti ME, Kuchel GA. Geriatric Syndromes: Clinical, Research, and Policy Implications of a Core Geriatric Concept. J Am Geriatr Soc 2007;55(5):780-791.

For peer review only - http://bmjopen.bmj.com/site/about/guidelines.xhtml

(7) Mudge AM, Banks MD, Barnett AG, Blackberry I, Graves N, Green T, et al. CHERISH (collaboration for hospitalised elders reducing the impact of stays in hospital): protocol for a multi-site improvement program to reduce geriatric syndromes in older inpatients. BMC Geriatrics 2017;17(1):11.
(8) Covinsky KE, Pierluissi E, Johnston CB. Hospitalization-associated disability: "She was probably able to ambulate, but I'm not sure". JAMA 2011 Oct 26;306(16):1782-1793.
(9) Zisberg A, Shadmi E, Gur-Yaish N, Tonkikh O, Sinoff G. Hospital-associated functional decline: the role of hospitalization processes beyond individual risk factors. J Am Geriatr Soc 2015 Jan;63(1):55-62.
(10) Fried LP, Tangen CM, Walston J, Newman AB, Hirsch C, Gottdiener J, et al. Frailty in older adults: evidence for a phenotype. J Gerontol A Biol Sci Med Sci 2001 Mar;56(3):M146-56.
(11) Clegg A, Young J, Iliffe S, Rikkert MO, Rockwood K. Frailty in elderly people. The Lancet 2013 3/2–8;381(9868):752-762.
(12) Ferrucci L, Guralnik JM, Studenski S, Fried LP, Cutler GB, Walston JD, et al. Designing Randomized, Controlled Trials Aimed at Preventing or Delaying Functional Decline and Disability in Frail, Older Persons: A Consensus Report. J Am Geriatr Soc 2004;52(4):625-634.
(13) Glover A, Bradshaw LE, Watson N, Laithwaite E, Goldberg SE, Whittamore KH, et al. Diagnoses, problems and healthcare interventions amongst older people with an unscheduled hospital admission who have concurrent mental health problems: a prevalence study. BMC geriatrics 2014;14(1):1.
(14) Pinkert C, Holle B. People with dementia in acute hospitals. Literature review of prevalence and reasons for hospital admission. Z Gerontol Geriatr 2012 Dec;45(8):728-734.
(15) Mukadam N, Sampson EL. A systematic review of the prevalence, associations and outcomes of dementia in older general hospital inpatients. International Psychogeriatrics 2011;23(03):344-355.
(16) Timmons S, Manning E, Barrett A, Brady NM, Browne V, O'Shea E, et al. Dementia in older people admitted to hospital: a regional multi-hospital observational study of prevalence, associations and case recognition. Age Ageing 2015 Nov;44(6):993-999.
(17) Bradshaw LE, Goldberg SE, Lewis SA, Whittamore K, Gladman JRF, Jones RG, et al. Six-month outcomes following an emergency hospital admission for older adults with co-morbid mental health problems indicate complexity of care needs. Age and Ageing 2013 September 01;42(5):582-588.
(18) Satizabal CL, Beiser AS, Chouraki V, Chêne G, Dufouil C, Seshadri S. Incidence of Dementia over Three Decades in the Framingham Heart Study. N Engl J Med 2016 02/11; 2016/02;374(6):523-532.
(19) World Health Organization. Dementia Fact sheet. April 2016; Available at: http://www.who.int/mediacentre/factsheets/fs362/en/. Accessed January 25, 2017.
(20) Fried TR, van Doorn C, O'Leary JR, Tinetti ME, Drickamer MA. Older persons' preferences for home vs hospital care in the treatment of acute illness. Arch Intern Med 2000;160(10):1501-1506.
(21) Fried TR, Bradley EH, Towle VR, Allore H. Understanding the Treatment Preferences of Seriously Ill Patients. N Engl J Med 2002 04/04; 2017/05;346(14):1061-1066.
(22) Shepperd S, Iliffe S, Doll HA, Clarke MJ, Kalra L, Wilson AD, et al. Admission avoidance hospital at home. The Cochrane Library 2016.
(23) Conley J, O'Brien CW, Leff BA, Bolen S,Zulman D. Alternative strategies to inpatient hospitalization for acute medical conditions: A systematic review. JAMA Internal Medicine 2016 November 1;176(11):1693-1702.
(24) Caplan G. Does 'Hospital in the Home' treatment prevent delirium? Aging Health 2008 02/01; 2017/12;4(1):69-74.
(25) Tibaldi V, Aimonino N, Ponzetto M, Stasi M, Amati D, Raspo S, et al. A randomized controlled trial of a home hospital intervention for frail elderly demented patients: behavioral disturbances and caregiver's stress. Arch Gerontol Geriatr 2004;38:431-436.
(26) Shepperd S, Cradduck-Bamford A, Butler C, Ellis G, Godfrey M, Gray A, et al. A multi-centre randomised trial to compare the effectiveness of geriatrician-led admission avoidance hospital at home versus inpatient admission. Trials 2017;18:10.1186/s13063-017-2214-y.
(27) Chan AW, Tetzlaff JM, Altman DG, Laupacis A, Gotzsche PC, Krleza-Jeric K, et al. SPIRIT 2013 statement: defining standard protocol items for clinical trials. Ann Intern Med 2013 Feb 5;158(3):200-207.
(28) Bellelli G, Morandi A, Davis DHJ, Mazzola P, Turco R, Gentile S, et al. Validation of the 4AT, a new instrument for rapid delirium screening: a study in 234 hospitalised older people. Age and Ageing 2014 March 02.
(29) Katzman R, Brown T, Fuld P, Peck A, Schechter R, Schimmel H. Validation of a short Orientation-Memory-Concentration Test of cognitive impairment. Am J Psychiatry 1983 Jun;140(6):734-739.
(30) Shepperd S, Harwood D, Jenkinson C, Gray A, Vessey M, Morgan P. Randomised controlled trial comparing hospital at home care with inpatient hospital care. I: three month follow up of health outcomes. BMJ 1998 Jun 13;316(7147):1786-1791.

For peer review only - http://bmjopen.bmj.com/site/about/guidelines.xhtml

(31) Cei M, Bartolomei C, Mumoli N. In-hospital mortality and morbidity of elderly medical patients can be predicted at admission by the Modified Early Warning Score: a prospective study. Int J Clin Pract 2009;63(4):591-595.
(32) Dundar ZD, Ergin M, Karamercan MA, Ayranci K, Colak T, Tuncar A, et al. Modified Early Warning Score and VitalPac Early Warning Score in geriatric patients admitted to emergency department. Eur J Emerg Med 2015.
(33) Ridley S. The recognition and early management of critical illness. Ann R Coll Surg Engl 2005 Sep;87(5):315-322.
(34) Aimonino Ricauda N, Tibaldi V, Leff B, Scarafiotti C, Marinello R, Zanocchi M, et al. Substitutive “hospital at home” versus inpatient care for elderly patients with exacerbations of chronic obstructive pulmonary disease: a prospective randomized, controlled trial. J Am Geriatr Soc 2008;56(3):493-500.
(35) Tibaldi V, Isaia G, Scarafiotti C, Gariglio F, Zanocchi M, Bo M, et al. Hospital at home for elderly patients with acute decompensation of chronic heart failure: a prospective randomized controlled trial. Arch Intern Med 2009;169(17):1569-1575.
(36) Worth A, Tierney AJ. Conducting research interviews with elderly people by telephone. J Adv Nurs 1993;18(7):1077-1084.
(37) Subbe CP, Kruger M, Rutherford P, Gemmel L. Validation of a modified Early Warning Score in medical admissions. QJM 2001 Oct;94(10):521-526.
(38) American College of Emergency Physicians, American Geriatrics Society, Emergency Nurses Association, Society for Academic Emergency Medicine, Geriatric Emergency Department Guidelines Task Force. Geriatric emergency department guidelines. Ann Emerg Med 2014 May;63(5):e7-25.
(39) Charlson ME, Pompei P, Ales KL, MacKenzie CR. A new method of classifying prognostic comorbidity in longitudinal studies: development and validation. J Chronic Dis 1987;40(5):373-383.
(40) Oud F, de Rooij S, Schuurman T, Duijvelaar K, van Munster B. [Predictive value of the VMS theme'Frail elderly': delirium, falling and mortality in elderly hospital patients]. Ned Tijdschr Geneeskd 2014;159:A8491-A8491.
(41) Katz S, Ford AB, Moskowitz RW, Jackson BA, Jaffe MW. Studies of illness in the aged: the index of ADL: a standardized measure of biological and psychosocial function. JAMA 1963;185(12):914-919.
(42) Herdman M, Gudex C, Lloyd A, Janssen M, Kind P, Parkin D, et al. Development and preliminary testing of the new five-level version of EQ-5D (EQ-5D-5L). Quality of Life Research 2011;20(10):1727-1736.
(43) Makai P, Brouwer WBF, Koopmanschap MA, Nieboer AP. Capabilities and quality of life in Dutch psycho-geriatric nursing homes: an exploratory study using a proxy version of the ICECAP-O. Quality of Life Research 2012;21(5):801-812.
(44) van Exel NJ, Scholte op Reimer WJ, Brouwer WB, van den Berg B, Koopmanschap MA, van den Bos GA. Instruments for assessing the burden of informal caregiving for stroke patients in clinical practice: a comparison of CSI, CRA, SCQ and self-rated burden. Clin Rehabil 2004 Mar;18(2):203-214.
(45) Robinson BC. Validation of a Caregiver Strain Index. Journal of Gerontology 1983 May 01;38(3):344-348.
(46) Bouwmans C, Hakkaart-van Roijen L, Koopmanschap M, Krol M, Severens H, Brouwer W. Handleiding iMTA Medical Cost Questionnaire (iMCQ). Rotterdam: iMTA, Erasmus Universiteit Rotterdam, 2013.
(47) Gavinski K, Carnahan R, Weckmann M. Validation of the delirium observation screening scale in a hospitalized older population. Journal of Hospital Medicine 2016:n/a-n/a.
(48) Stratton RJ, Hackston A, Longmore D, Dixon R, Price S, Stroud M, et al. Malnutrition in hospital outpatients and inpatients: prevalence, concurrent validity and ease of use of the ‘malnutrition universal screening tool’(‘MUST’) for adults. Br J Nutr 2004;92(05):799-808.
(49) Hawker GA, Mian S, Kendzerska T, French M. Measures of adult pain: Visual Analog Scale for Pain (VAS Pain), Numeric Rating Scale for Pain (NRS Pain), McGill Pain Questionnaire (MPQ), Short-Form McGill Pain Questionnaire (SF-MPQ), Chronic Pain Grade Scale (CPGS), Short Form-36 Bodily Pain Scale (SF-36 BPS), and Measure of Intermittent and Constant Osteoarthritis Pain (ICOAP). Arthritis Care & Research 2011;63(S11):S240-S252.
(50) de Haes,J.C., van Knippenberg,F.C., Neijt JP. Measuring psychological and physical distress in cancer patients: structure and application of the Rotterdam Symptom Checklist. Br J Cancer 1990 12;62(6):1034-1038.
(51) MacKnight C, Rockwood K. A Hierarchical Assessment of Balance and Mobility. Age Ageing 1995 Mar;24(2):126-130.
(52) Attkisson CC, Greenfield TK. The UCSF Client Satisfaction Scales: I. The Client Satisfaction Questionnaire-8. 1999.
(53) Reichheld FF. The one number you need to grow. Harv Bus Rev 2003;81(12):46-55.
(54) Quinn TJ, Dawson J, Lees JS, Chang T, Walters MR, Lees KR, et al. Time Spent at Home Poststroke: “Home-Time” a Meaningful and Robust Outcome Measure for Stroke Trials. Stroke 2008 January 01;39(1):231-233.

For peer review only - http://bmjopen.bmj.com/site/about/guidelines.xhtml

(55) Drummond MF, Sculpher MJ, Claxton K, Stoddart GL, Torrance GW. Methods for the economic evaluation of health care programmes. : Oxford university press; 2015.
(56) Hakkaart-van Roijen L, Van der Linden N, Bouwmans C, Kanters T, Tan S. Kostenhandleiding. Methodologie van kostenonderzoek en referentieprijzen voor economische evaluaties in de gezondheidszorg. In opdracht van Zorginstituut Nederland. Geactualiseerde versie 2015.
(57) Ritchie J, Spencer L. Qualitative data analysis for applied policy research. The qualitative researcher's companion 2002;573(2002):305-329.
(58) Wet bescherming persoonsgegevens (2000, 6th of July). Available at: http://wetten.overheid.nl/BWBR0011468/2017-07-01/0/. Accessed October 25, 2017.
(59) Schulz KF, Altman DG, Moher D. CONSORT 2010 Statement: updated guidelines for reporting parallel group randomised trials. Trials 2010;11(1):32.
(60) Raad voor de leefomgeving en infrastructuur. Langer zelfstandig, een gedeelde opgave van wonen, zorg en welzijn. Advies van de Raad voor de Leefomgeving en infrastructuur over langer zelfstandig wonen, 2014. Available at http://www.rli.nl/sites/default/files/rli-briefadvieslangerzelfstandigeengedeeldeopgavevanwonenzorgenwelzijndef23-113-30_0.pdf Accessed October 25, 2017.
(61) Caplan GA, Sulaiman NS, Mangin DA, Aimonino Ricauda N, Wilson AD, Barclay L. A meta-analysis of "hospital in the home". Med J Aust 2012 Nov 5;197(9):512-519.
(62) Porter ME. What Is Value in Health Care? N Engl J Med 2010 12/23; 2016/06;363(26):2477-2481.
(63) Bradshaw LE, Goldberg SE, Lewis SA, Whittamore K, Gladman J, Jones R, et al. Six-month outcomes following an emergency hospital admission for older adults with co-morbid mental health problems indicate complexity of care needs. Age Ageing 2013;42(5):582-588.
(64) Sayer C. "Time Spent at Home" — A Patient-Defined Outcome. NEJM Catalyst. 2016.
(65) Groff AC, Colla CH, Lee TH. Days Spent at Home — A Patient-Centered Goal and Outcome. N Engl J Med 2016 10/27; 2017/01;375(17):1610-1612.

Figure Legends

Figure 1: Flowchart of trial design summary

For peer review only - http://bmjopen.bmj.com/site/about/guidelines.xhtml

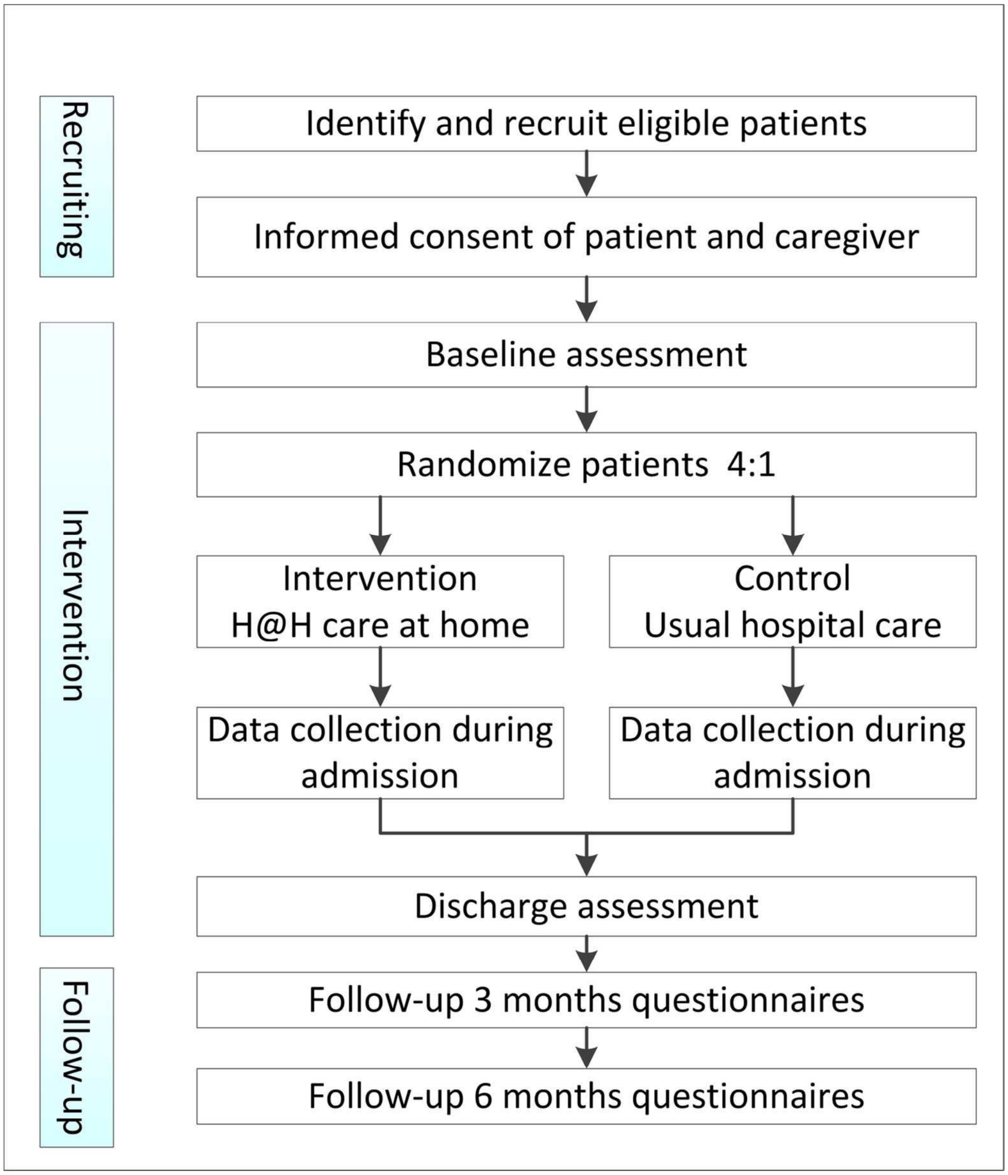

Figure 1: Flowchart of trial design summary

127x148mm (300 x 300 DPI)

SPIRIT

STANDARD PROTOCOL ITEMS: RECOMMENDATIONS FOR INTERVENTIONAL TRIALS

SPIRIT 2013 Checklist: Recommended items to address in a clinical trial protocol and related documents*

Section/item	Item No	Description	Addressed on page number
**Administrative information**			
Title	1	Descriptive title identifying the study design, population, interventions, and, if applicable, trial acronym	1
Trial registration	2a	Trial identifier and registry name. If not yet registered, name of intended registry	1
	2b	All items from the World Health Organization Trial Registration Data Set	1, 3, 4, 5, 6, 8, 9, 13, 14
Protocol version	3	Date and version identifier	all
Funding	4	Sources and types of financial, material, and other support	14
Roles and responsibilities	5a	Names, affiliations, and roles of protocol contributors	14
	5b	Name and contact information for the trial sponsor	14
	5c	Role of study sponsor and funders, if any, in study design; collection, management, analysis, and interpretation of data; writing of the report; and the decision to submit the report for publication, including whether they will have ultimate authority over any of these activities	14
	5d	Composition, roles, and responsibilities of the coordinating centre, steering committee, endpoint adjudication committee, data management team, and other individuals or groups overseeing the trial, if applicable (see Item 21a for data monitoring committee)	n/a

For peer review only - http://bmjopen.bmj.com/site/about/guidelines.xhtml

**Introduction**			
Background and rationale	6a	Description of research question and justification for undertaking the trial, including summary of relevant studies (published and unpublished) examining benefits and harms for each intervention	3
	6b	Explanation for choice of comparators	3
Objectives	7	Specific objectives or hypotheses	3
Trial design	8	Description of trial design including type of trial (eg, parallel group, crossover, factorial, single group), allocation ratio, and framework (eg, superiority, equivalence, noninferiority, exploratory)	4
**Methods: Participants, interventions, and outcomes**			
Study setting	9	Description of study settings (eg, community clinic, academic hospital) and list of countries where data will be collected. Reference to where list of study sites can be obtained	4
Eligibility criteria	10	Inclusion and exclusion criteria for participants. If applicable, eligibility criteria for study centres and individuals who will perform the interventions (eg, surgeons, psychotherapists)	6
Interventions	11a	Interventions for each group with sufficient detail to allow replication, including how and when they will be administered	7, 8
	11b	Criteria for discontinuing or modifying allocated interventions for a given trial participant (eg, drug dose change in response to harms, participant request, or improving/worsening disease)	7, 8
	11c	Strategies to improve adherence to intervention protocols, and any procedures for monitoring adherence (eg, drug tablet return, laboratory tests)	n/a
	11d	Relevant concomitant care and interventions that are permitted or prohibited during the trial	9, 10
Outcomes	12	Primary, secondary, and other outcomes, including the specific measurement variable (eg, systolic blood pressure), analysis metric (eg, change from baseline, final value, time to event), method of aggregation (eg, median, proportion), and time point for each outcome. Explanation of the clinical relevance of chosen efficacy and harm outcomes is strongly recommended	9, 10
Participant timeline	13	Time schedule of enrolment, interventions (including any run-ins and washouts), assessments, and visits for participants. A schematic diagram is highly recommended (see Figure)	10

For peer review only - http://bmjopen.bmj.com/site/about/guidelines.xhtml

Sample size	14	Estimated number of participants needed to achieve study objectives and how it was determined, including clinical and statistical assumptions supporting any sample size calculations	6
Recruitment	15	Strategies for achieving adequate participant enrolment to reach target sample size	5
**Methods: Assignment of interventions (for controlled trials)**			
Allocation:			
Sequence generation	16a	Method of generating the allocation sequence (eg, computer-generated random numbers), and list of any factors for stratification. To reduce predictability of a random sequence, details of any planned restriction (eg, blocking) should be provided in a separate document that is unavailable to those who enrol participants or assign interventions	4
Allocation concealment mechanism	16b	Mechanism of implementing the allocation sequence (eg, central telephone; sequentially numbered, opaque, sealed envelopes), describing any steps to conceal the sequence until interventions are assigned	4
Implementation	16c	Who will generate the allocation sequence, who will enrol participants, and who will assign participants to interventions	4
Blinding (masking)	17a	Who will be blinded after assignment to interventions (eg, trial participants, care providers, outcome assessors, data analysts), and how	4
	17b	If blinded, circumstances under which unblinding is permissible, and procedure for revealing a participant's allocated intervention during the trial	n/a
**Methods: Data collection, management, and analysis**			
Data collection methods	18a	Plans for assessment and collection of outcome, baseline, and other trial data, including any related processes to promote data quality (eg, duplicate measurements, training of assessors) and a description of study instruments (eg, questionnaires, laboratory tests) along with their reliability and validity, if known. Reference to where data collection forms can be found, if not in the protocol	9, 10, 11
	18b	Plans to promote participant retention and complete follow-up, including list of any outcome data to be collected for participants who discontinue or deviate from intervention protocols	11

For peer review only - http://bmjopen.bmj.com/site/about/guidelines.xhtml

Data management	19	Plans for data entry, coding, security, and storage, including any related processes to promote data quality (eg, double data entry; range checks for data values). Reference to where details of data management procedures can be found, if not in the protocol	11
Statistical methods	20a	Statistical methods for analysing primary and secondary outcomes. Reference to where other details of the statistical analysis plan can be found, if not in the protocol	12
	20b	Methods for any additional analyses (eg, subgroup and adjusted analyses)	n/a
	20c	Definition of analysis population relating to protocol non-adherence (eg, as randomised analysis), and any statistical methods to handle missing data (eg, multiple imputation)	n/a
**Methods: Monitoring**			
Data monitoring	21a	Composition of data monitoring committee (DMC); summary of its role and reporting structure; statement of whether it is independent from the sponsor and competing interests; and reference to where further details about its charter can be found, if not in the protocol. Alternatively, an explanation of why a DMC is not needed	12
	21b	Description of any interim analyses and stopping guidelines, including who will have access to these interim results and make the final decision to terminate the trial	12
Harms	22	Plans for collecting, assessing, reporting, and managing solicited and spontaneously reported adverse events and other unintended effects of trial interventions or trial conduct	12
Auditing	23	Frequency and procedures for auditing trial conduct, if any, and whether the process will be independent from investigators and the sponsor	n/a
**Ethics and dissemination**			
Research ethics approval	24	Plans for seeking research ethics committee/institutional review board (REC/IRB) approval	12
Protocol amendments	25	Plans for communicating important protocol modifications (eg, changes to eligibility criteria, outcomes, analyses) to relevant parties (eg, investigators, REC/IRBs, trial participants, trial registries, journals, regulators)	12

For peer review only - http://bmjopen.bmj.com/site/about/guidelines.xhtml

Consent or assent	26a	Who will obtain informed consent or assent from potential trial participants or authorised surrogates, and how (see Item 32)	5
	26b	Additional consent provisions for collection and use of participant data and biological specimens in ancillary studies, if applicable	n/a
Confidentiality	27	How personal information about potential and enrolled participants will be collected, shared, and maintained in order to protect confidentiality before, during, and after the trial	10
Declaration of interests	28	Financial and other competing interests for principal investigators for the overall trial and each study site	13
Access to data	29	Statement of who will have access to the final trial dataset, and disclosure of contractual agreements that limit such access for investigators	10
Ancillary and post-trial care	30	Provisions, if any, for ancillary and post-trial care, and for compensation to those who suffer harm from trial participation	Available on request
Dissemination policy	31a	Plans for investigators and sponsor to communicate trial results to participants, healthcare professionals, the public, and other relevant groups (eg, via publication, reporting in results databases, or other data sharing arrangements), including any publication restrictions	12
	31b	Authorship eligibility guidelines and any intended use of professional writers	13
	31c	Plans, if any, for granting public access to the full protocol, participant-level dataset, and statistical code	n/a
**Appendices**			
Informed consent materials	32	Model consent form and other related documentation given to participants and authorised surrogates	Available on request
Biological specimens	33	Plans for collection, laboratory evaluation, and storage of biological specimens for genetic or molecular analysis in the current trial and for future use in ancillary studies, if applicable	n/a

*It is strongly recommended that this checklist be read in conjunction with the SPIRIT 2013 Explanation & Elaboration for important clarification on the items. Amendments to the protocol should be tracked and dated. The SPIRIT checklist is copyrighted by the SPIRIT Group under the Creative Commons "Attribution-NonCommercial-NoDerivs 3.0 Unported" license.

For peer review only - http://bmjopen.bmj.com/site/about/guidelines.xhtml
